# An open-label, randomized, non-inferiority trial of the efficacy and safety of ciprofloxacin versus streptomycin + ciprofloxacin in the treatment of bubonic plague (IMASOY): study protocol for a randomized control trial

**DOI:** 10.1186/s13063-020-04642-2

**Published:** 2020-08-17

**Authors:** Rindra Vatosoa Randremanana, Mihaja Raberahona, Mamy Jean de Dieu Randria, Minoarisoa Rajerison, Voahangy Andrianaivoarimanana, Agathe Legrand, Tsinjo Fehizoro Rasoanaivo, Ravaka Randriamparany, Théodora Mayouya-Gamana, Reziky Mangahasimbola, Josie Bourner, Alex Salam, Annelies Gillesen, Tansy Edwards, Matthieu Schoenhals, Laurence Baril, Peter Horby, Piero Olliaro

**Affiliations:** 1 Institut Pasteur de Madagascar, Antananarivo, Madagascar; 2 Infectious Diseases Department, University Hospital Joseph Raseta Befelatanana Antananarivo – Centre d’Infectiologie Charles Mérieux, University of Antananarivo, Antananarivo, Madagascar; 3 Infectious Diseases Department, University Hospital Joseph Raseta, Antananarivo, Madagascar; 4 University of Oxford, Oxford, UK; 5 London School of Hygiene and Tropical Medicine, London, UK

**Keywords:** Plague, Bubonic plague, Pneumonic plague, Streptomycin, Ciprofloxacin

## Abstract

**Background:**

Bubonic plague is the primary manifestation of infection with *Yersinia pestis*, accounting for 90% of all plague cases and with 75% of global cases reported in Madagascar. All drugs in use for treating plague are registered based on experimental data and anecdotal evidence, and no regimen currently recommended is supported by a randomized clinical trial. The IMASOY trial intends to fill this knowledge gap by comparing two 10-day regimens included in the national guidelines in Madagascar. The primary objective of the trial is to test the hypothesis that ciprofloxacin monotherapy is non-inferior to streptomycin followed by ciprofloxacin for the treatment of bubonic plague, thus avoiding the need for injectable, potentially toxic, aminoglycosides.

**Methods:**

A two-arm parallel-group randomized control trial will be conducted across peripheral health centres in Madagascar in five districts. Males and non-pregnant females of all ages with suspected bubonic or pneumonic plague will be recruited over the course of three plague ‘seasons’. The primary endpoint of the trial is to assess the proportion of patients with bubonic plague who have a therapeutic response to treatment (defined as alive, resolution of fever, 25% reduction in the size of measurable buboes, has not received an alternative treatment and no clinical decision to continue antibiotics) as assessed on day 11.

**Discussion:**

If successful, the trial has the potential to inform the standard of care guidelines not just in Madagascar but in other countries afflicted by plague. The trial is currently ongoing and expected to complete recruitment in 2022.

**Trial registration:**

ClinicalTrials.gov NCT04110340. Registered on 1 October 2019

## Administrative information

**Table:** 

Title {1}	An open-label, randomized, non-inferiority trial of the efficacy and safety of ciprofloxacin versus streptomycin + ciprofloxacin in the treatment of bubonic plague (IMASOY)
Trial registration {2a and 2b}.	ClinicalTrials.gov, NCT04110340, registered 01-Oct-2019, https://clinicaltrials.gov/ct2/show/NCT04110340
Protocol version {3}	v2.8 27-Sep-2019
Funding {4}	Wellcome Trust/Department for International Development (WT/DFID)
Author details {5a}	Rindra Vatosoa Randremanana – Institut Pasteur de Madagascar Mihaja Raberahona – Infectious Diseases Department, University Hospital Joseph Raseta Befelatanana Antananarivo – Centre d’Infectiologie Charles Mérieux, University of Antananarivo Mamy Jean de Dieu Randria – Infectious Diseases Department, University Hospital Joseph Raseta Befelatanana Antananarivo Minoarisoa Rajerison - Institut Pasteur de Madagascar Voahangy Andrianaivoarimanana- Institut Pasteur de Madagascar Agathe Legrand – Institut Pasteur de Madagascar Tsinjo Fehizoro Rasoanaivo – Institut Pasteur de Madagascar Ravaka Randriamparany – Institut Pasteur de Madagascar Théodora Mayouya-Gamana – Institut Pasteur de Madagascar Reziky Mangahasimbola – Institut Pasteur de Madagascar Josie Bourner – University of Oxford Alex Salam – University of Oxford Annelies Gillesen – University of Oxford Tansy Edwards – London School of Hygiene and Tropical Medicine Matthieu Schoenhals- Institut Pasteur de Madagascar Laurence Baril- Institut Pasteur de Madagascar Peter Horby – University of Oxford Piero Olliaro – University of Oxford
Name and contact information for the trial sponsor {5b}	University of Oxford Joint Research Office 1st floor, Boundary Brook House Churchill Drive, Headington Oxford OX3 7GB, UK Trial Manager email address: josephine.bourner@ndm.ox.ac.uk
Role of sponsor {5c}	In collaboration, the University of Oxford, Institut Pasteur de Madagascar (IPM), University Hospital Joseph Raseta Befelatanana Antananarivo, Centre Infectiologie Charles Mérieux Madagascar are responsible for the design and management of the IMASOY trial. The University of Oxford and IPM are jointly responsible for data collection and management. All collaborators will be responsible for writing and publishing the final report. The London School of Hygiene and Tropical Medicine is primarily responsible for the statistical analysis, alongside IPM de Madagascar and the University of Oxford. Any further publications that arise following the dissemination of the final report will be reviewed by the University of Oxford before their release. All publications will be open-access. Laboratory data analysis will be the responsibility of IPM.

## Introduction

### Background and rationale {6a}

#### Plague

Plague is caused by the Gram-negative bacteria *Yersinia pestis*. There are three main clinical forms in which plague can manifest, sometimes with overlapping clinical syndromes: bubonic plague, septicaemic plague (primary or secondary) and pneumonic plague (primary or secondary) [1]. Bubonic plague is the most common clinical form and is characterized by the rapid onset of fever and tender and painful lymphadenopathy. It accounts for over 90% of plague cases [1]. Septicaemic plague is characterized by non-specific symptoms, as for septicaemia in general [1]. Pneumonic plague presents with rapid onset of fever and respiratory symptoms, with haemoptysis being a cardinal feature [1].

Plague is primarily present in Africa, although the USA and countries in Asia and South America are also affected. The incidence of plague continues to decrease (from 2683 cases notified to the WHO in 2008 to 243 in 2018) and geographical spread to shrink (98% of reported cases currently from just Madagascar and the Democratic Republic of Congo (DRC)) [2]. However, the reservoir and conditions for transmission are still present in many areas of the world, and there is a risk of outbreaks when plague hits densely populated areas, as in the 2017 large urban outbreak of predominantly pneumonic plague in Madagascar, with over 2400 suspected cases [3].

In the pre-antibiotic era, the case fatality rate (CFR) of plague was > 70% [4]. Antibiotics have significantly reduced mortality: for the cases notified to WHO during 2010–2018 CFR averaged 17% [2]. During the 2017–2018 plague outbreak in Madagascar, the CFR of confirmed pneumonic plague was 25% and bubonic plague 24% [3].

#### Current treatment options

Until July 2018, the 1st-line treatment regimen for bubonic plague in Madagascar was streptomycin (days 1 to 5) followed by cotrimoxazole (days 3 to 8). Following the 2017 epidemic, an After Action Review was conducted and treatment guidelines for bubonic plague were modified to include, for adults, either streptomycin intramuscularly 1 g (except pregnant women) or gentamicin 2.5 mg/kg twice daily for 3 days, followed by ciprofloxacin 500 mg twice daily for an additional 7 days or ciprofloxacin alone at 500 mg twice daily for 10 days. Intravenous ciprofloxacin at a dose of 400 mg every 12 h can be used if the oral route is not possible. Treatment options in children are now either oral ciprofloxacin 15 mg/kg twice daily for 10 days or, if oral administration is not initially possible, streptomycin 15 mg/kg every 12 h intramuscularly for 3 days followed by ciprofloxacin 15 mg/kg twice daily for an additional 7 days.

The first-line treatment regimen for pneumonic plague in adults is streptomycin 1 g intramuscularly twice daily for 5 days combined with ciprofloxacin 500 mg orally twice daily for 10 days, and for children, intravenous gentamicin at 2.5 mg/kg every 12 h for 5 days (or streptomycin) combined with oral ciprofloxacin at 15 mg/kg every 12 h for 10 days (with the possibility of intravenous ciprofloxacin at 15 mg/kg every 12 h if the oral route is not available). The maximum dose for ciprofloxacin is 500 mg per oral dose and 400 mg per intravenous dose for children.

Streptomycin alone or in other combination therapies is also the treatment of choice in other countries, including the DRC and Peru.

#### Study rationale

The evidence base for the current treatment regimens for plague is weak, and none is supported by a randomized clinical trial (RCT). Two randomized control trials have previously attempted to improve on or contribute to a better evidence base for current plague treatment regimens [5, 6]. However, neither trial was successful nor included children under 8 years of age, who constitute a significant proportion of the population affected by plague. Treatment recommendations are based on animal models, case reports, case series and a trial of doxycycline versus gentamicin [5]. All drugs approved for treating plague by the the United States Food and Drug Administration (US FDA) (streptomycin, tetracyclines and the fluoroquinolones ciprofloxacin, levofloxacin and moxifloxacin) are registered based on the ‘animal efficacy rule’, in the absence of clinical data [7]. Political instability and practical difficulties related to highly dispersed cases in rural areas have been the major obstacles to conducting treatment trials of plague.

The main issue with current regimens in Madagascar is the use of aminoglycosides—with the need for injections, class toxicity and need for monitoring of renal and auditory functions, which is not routinely available in most low- and middle-income countries, and streptomycin cost and stock-outs.

Ciprofloxacin is cheaper, more readily available in low- and middle-income countries, has greater ease of administration and there is no need for biochemical or drug monitoring. There is also no reason to believe that there is a difference in effectiveness. These reasons would likely result in ciprofloxacin being the first-line choice for the treatment of plague should it be shown to be effective.

### Objectives {7}

The primary objective of this trial is to test the hypothesis that ciprofloxacin monotherapy given (orally, or for subjects who cannot take oral medications intravenously or in combination) for 10 days is non-inferior to streptomycin (given on days 1–3) followed by ciprofloxacin (given on days 4–10) for the treatment of bubonic plague. The regimens are respectively third- and first-line treatments as per the national plague treatment guidelines in Madagascar.

The secondary objective is to collect data on the effectiveness of ciprofloxacin in the treatment of pneumonic plague, although the trial is not able to formally assess the non-inferiority of ciprofloxacin monotherapy compared to streptomycin and ciprofloxacin combination therapy in pneumonic plague,

Considering the operational and practical complexities of a plague RCT, the study also has additional exploratory objectives to optimize investments: to evaluate the level and kinetics of anti-*Y. pestis* antibodies in bubonic and pneumonic plague during treatment and follow-up, to compare different methods for detection of anti-*Y. pestis* antibodies, to measure the performance of qPCR for plague diagnosis and to allow for the evaluation of new rapid tests that may become available.

### Trial design {8}

#### Study schematics

This is an individually randomized two-group parallel arm control trial with a 1:1 ratio. Patients will be randomized to receive either streptomycin + ciprofloxacin or ciprofloxacin alone. We are using a non-inferiority design since the overall cure rate for bubonic plague without septicaemia with streptomycin is expected to be 90%. As a result, demonstrating superiority would be unnecessary and impractical given the sample size that would be required. Our aim is therefore to demonstrate that ciprofloxacin alone is not more than 15% inferior to streptomycin followed by ciprofloxacin (15% is the non-inferiority margin in our study, meaning that the lower bound of the confidence interval around the risk difference in the success rates of streptomycin + ciprofloxacin and ciprofloxacin alone must not include 15%). The trial is powered for bubonic plague, although we will recruit patients with pneumonic plague as well.

#### Box 1—Case definitions

**Table:** 

Probable and confirmed cases are defined as:	
Probable case: rapid diagnostic test (RDT) or qPCR or serology (anti-F1 IgG ELISA) is positive but without evidence of seroconversion or a fourfold increase in antibody titre.	
Confirmed case: RDT and qPCR are positive, or culture is positive, or there has been a seroconversion or a fourfold increase in antibody titre on two separate serological samples (either between D1 and D11, between D11 and D21 or between D1 and D21).	

We will recruit patients with clinical suspicion of bubonic plague, but the size of our sample is powered based on an intention to treat infected patient sample size of 190, where ‘infected’ is defined as a ‘confirmed’ or ‘probable’ case of bubonic plague. As a result, the total number of patients to be enrolled will be higher than 190. We estimate that we will need to recruit approximately 600 patients with bubonic plague to achieve a sample size of 190 confirmed/probable bubonic plague patients. However, to mitigate risks of being under-powered, we will recruit for three full seasons (2019–2022) with a minimum target of 190 confirmed/probable cases, 95 patients in each arm.

Should we achieve the target of 190 confirmed/probable bubonic plague cases before the end of the third season (2019–2022), we will nevertheless continue to recruit until the end of the season to retain power in the event the observed treatment success rates differ from those expected, and to allow us to increase precision.

Whilst we will also recruit and collect data on patients with pneumonic plague, it is highly unlikely that we will have the power to complete a non-inferiority trial for ciprofloxacin monotherapy for pneumonic plague patients. For example, with a case fatality rate of 20–25%, we would need a sample size of approximately 400 patients with probable/confirmed pneumonic plague for a non-inferiority margin of 15%, which is unrealistic.

#### Study timelines

Recruitment is expected to last 3 years and will take place during each ‘plague season’ (the annual high transmission period of plague, which starts at the beginning of August and runs until the following March).

#### Study population

The study will enrol males and non-pregnant females of any age with suspected bubonic or pneumonic plague.

## Methods: participants, interventions and outcomes

### Study setting {9}

#### Study setting

The trial will take place in up to five pre-identified districts in Madagascar. Madagascar reports more plague cases per year than any other country with an average number of confirmed/probable cases per year of 387 [8].

#### Study sites

Recruitment is planned to take place in 50 health centres (primary health centres (Centres de Santé de Base, CSB) and district hospitals) in five districts. Recruiting districts and sites have been selected based on their incidence of plague (as reported to IPM) and also for logistical reasons. However, the trial may be carried out in further districts or sites depending on the real-time incidence of suspected cases of plague during the study period. The majority of plague patients are treated at the CSB level. In general, CSBs do not have a laboratory, so sites perform an RDT to obtain a preliminary diagnosis and then send further samples to IPM for confirmation where a second RDT, qPCR and culture are routinely performed.

### Eligibility criteria {10}

#### Inclusion criteria

##### Bubonic plague

- Patients of any age
- Recent onset (< 10 days) of fever (uncorrected axillary temperature ≥ 37.5 °C) or history of fever
- One or more buboes (tender lymph node swelling)
- Residence or travel to a plague endemic or outbreak area in Madagascar within 14 days of the onset of symptoms
- Patients identified as clinically suspected of plague by health personnel (doctors or paramedics)

##### Pneumonic plague

- Patients of any age
- Recent onset (< 7 days) of fever (uncorrected axillary temperature ≥ 37.5 °C) or history of fever
- Cough
- Tachypnoea (respiratory rate > 24 in adults and age-specific in children)
- Epidemiological link with a confirmed or probable case of primary or secondary pneumonic plague within 7 days of onset of symptoms

A small proportion of patients with bubonic plague will also develop pneumonic plague. These patients will be randomized to receive pneumonic plague therapy. For a patient to be considered to have mixed bubonic and pneumonic plague, they must have developed cough and dyspnoea after the onset of the bubo.

#### Exclusion criteria

- Known allergy to aminoglycosides or fluoroquinolones
- Tendinitis
- Myasthenia gravis
- Theophylline or warfarin use
- Already treated for bubonic or pneumonic plague in the preceding 3 months
- Women who report being pregnant

Given that streptomycin is an FDA class D drug, women who report being pregnant will not be randomized into the trial but will be treated outside the trial according to the national guidelines. Lactating women can safely be treated with streptomycin as streptomycin poses minimal risk to the infant when used during breastfeeding and will therefore be randomized into the trial.

### Who will take informed consent? {26a}

All eligible patients will be offered the opportunity to participate in the study. Patient information sheets, consent and assent forms will be available in relevant written local languages. Written informed consent to participate will be required from all participants or their representatives and will be requested by local study staff, who are qualified, trained and authorized to do so by the principal investigator.

### Additional consent provisions for collection and use of participant data and biological specimens {26b}

Patients are fully informed about the use of biological specimens and their data in the patient information sheet, including lay descriptions of the procedures for collecting samples and details about data and sample storage.

## Interventions

### Explanation for the choice of comparators {6b}

Both treatment regimens tested are provided for by the national guidelines. There are several reasons why streptomycin is a suboptimal therapeutic agent for plague including global stock shortages, the requirement for parenteral administration and severe toxicity.

In vitro assays suggest that ciprofloxacin has comparable efficacy to streptomycin and is superior to doxycycline or gentamicin for the killing of intracellular *Y. pestis* [6]. Studies in non-human primates of pneumonic and bubonic plague have also shown high therapeutic efficacy of ciprofloxacin, equivalent to streptomycin [9, 10]. Ciprofloxacin also offers the advantage of the possibility of a switch from intravenous to oral treatment if clinically appropriate, does not require drug level or renal function monitoring, has fewer and less severe side effects, it is classed by the US FDA as pregnancy category C (whereas other treatment alternatives are category D), has greater global availability and is cheaper. Unlike other treatment alternatives, ciprofloxacin can be administered to children.

### Intervention description {11a}

#### Dosing schedule in bubonic plague

##### Ciprofloxacin arm

- Adults: 500 mg orally twice daily (or 400 mg IV twice daily for those who cannot take oral medication) for 10 days.
- Children: 15 mg/kg twice daily (max 500 mg per dose) orally (or 10 mg/kg IV twice daily for those who cannot take oral—maximum dose 400 mg) for 10 days.
- Patients who begin intravenous therapy may switch to oral administration once they are able to swallow or once deemed clinically appropriate by the treating physician.

##### Control arm

- Adults: streptomycin 1 g twice daily for 3 days, followed by ciprofloxacin 500 mg orally twice daily (or ciprofloxacin 400 mg twice daily by IV for those who cannot take it orally) for an additional 7 days.
- Children: streptomycin 15 mg/kg twice daily for three days followed by ciprofloxacin 15 mg/kg twice daily (max 500 mg per dose) orally (or 10 mg/kg IV twice daily for those who cannot take oral—maximum dose 400 mg) for 7 additional days.
- Patients who start taking intravenous ciprofloxacin may switch to oral administration once they are able to swallow or once deemed clinically appropriate by the treating physician.

#### Dosing schedule in pneumonic plague

##### Ciprofloxacin arm

- Adults: 500 mg orally twice daily (or 400 mg IV twice daily for those who cannot take oral medication) for 10 days.
- Children: 15 mg/kg twice daily (max 500 mg per dose) orally (or 10 mg/kg IV twice daily for those who cannot take oral—maximum dose 400 mg) for 10 days.
- Patients who begin intravenous therapy may switch to oral administration once they are able to swallow or once deemed clinically appropriate by the treating physician.

##### Control arm

- Adults: streptomycin 1 g twice daily for 5 days with ciprofloxacin 500 mg orally twice daily (or ciprofloxacin 400 mg twice daily IV for those who cannot take it orally) for 10 days.
- Children: streptomycin 15 mg/kg twice daily for 5 days with ciprofloxacin 15 mg/kg twice daily (max 500 mg per dose) orally (or 10 mg/kg IV twice daily for those who cannot take it orally—maximum dose 400 mg) for 10 days. This is the alternative treatment proposed by the Ministry of Public Health since the 2018–2019 plague season.
- Patients who start taking intravenous ciprofloxacin may switch to oral administration once they are able to swallow or once deemed clinically appropriate by the treating physician.

#### Route of administration

Streptomycin is administered intramuscularly to all patients in the control group. Ciprofloxacin is administered orally, except for patients with the following manifestations:

- Vomiting
- Unable to swallow
- Unconscious
- Systolic blood pressure < 90 mmHg
- Any other conditions or situations for which the physician decides that intravenous treatment is indicated

#### Timing of dosing

- Ciprofloxacin: doses will be administered morning and evening.
- Streptomycin: doses will be administered morning and evening.

#### Duration of therapy

The study drugs will be administered for a total of 10 days. After 10 days, the decision whether to continue therapy will be at the treating physician’s discretion.

#### Dosing duration justification

We are using 10 days of therapy in both the ciprofloxacin arm and streptomycin and ciprofloxacin arm as this is the current standard duration of plague treatment in Madagascar. To date, the only RCT in plague used 7 days of therapy (gentamicin versus doxycycline) in both groups.

#### Prohibited drugs

- Theophylline
- Warfarin

### Criteria for discontinuing or modifying allocated interventions {11b}

If, for any reason, a dose of the study drug is not administered at the scheduled time, it may be administered later, but not more than 6 h after the scheduled time of administration. No dose adjustment is planned.

### Strategies to improve adherence to interventions {11c}

The trial will be co-managed by IPM who will deploy a clinical research associate and two clinical study nurse to each district to assist with the recruitment, trial procedures and data entry. These members of staff will be based in the districts for the duration of each season.

Before activation, site investigators will receive training on the identification of plague patients and the study protocol. After activation, the site investigators will be responsible for patient management alongside the clinical study nurse.

To optimize patient compliance, community health workers (CHWs) in each district will be trained to identify cases of plague based on community case definition as per the national guideline, refer patients to the local CSB I/II, perform treatment visits to patient homes and complete the treatment record for each home-based visit. At the time of discharge from the facility, patients will also be equipped with a mobile phone, as follow-up visits on D11 and D21, and possibly M3 will require a return visit to the treating CSB.

At the beginning of each new plague season, all site staff will be retrained on the trial protocol and reinitiated to the trial. This will reinforce the protocol following the annual recruitment suspension and ensure that, due to high staff turnover at the CSBs, any new staff members receive the same comprehensive training as those who were trained at the original initiation visit.

A monitoring plan has been developed to ensure regular reviews of trial data and site compliance with the protocol. These reviews will be completed within 72 h of each of the first five patients being recruited at each site, annually at the end of each plague season and on the identification of persistent or serious protocol violations and serious breaches. The Trial Operations Committee (TOC) will also meet on a weekly basis to review recruitment and compliance and discuss any other issues affecting the trial.

The trial has also appointed a Data Safety Monitoring Board (DSMB) and Trial Steering Committee, who will meet regularly to discuss the management of the trial.

### Relevant concomitant care permitted or prohibited during the trial {11d}

Other treatments, including the administration of fluids and organ support, will be at the discretion of the treating clinician.

### Provisions for post-trial care {30}

#### Post-trial care

Following their exit from the trial, patients will return to their standard care pathway.

#### Insurance

The University of Oxford, as study sponsor, has arranged appropriate insurances to provide for the University’s responsibilities to research subjects and to cover the legal liabilities of the University to those engaged by the University in the performance of this research.

### Outcomes {12}

#### Primary endpoint

The proportion of patients with bubonic plague with a therapeutic response (assessed on D11). Therapeutic response is defined as follows for subjects with a visible and measurable bubo:

- Alive
- Resolution of fever (uncorrected axillary temperature < 37.5 °C)
- A ≥ 25% decrease in bubo size (in the case of multiple buboes, the largest bubo)
- Has not received alternative treatment for plague
- No clinical decision to continue anti-plague antibiotics beyond day 10

For patients with small buboes that are palpable but not measurable:

- Alive
- Absence of fever (uncorrected axillary temperature < 37.5 °C)
- Bubo has not enlarged
- Has not received alternative treatment for plague
- No clinical decision to continue anti-plague antibiotics beyond day 10

#### Secondary endpoints

##### Bubonic plague

- Proportion of patients without fever (uncorrected axillary temperature < 37.5 °C) at D4
- Proportion of patients with a pain score < 3 at D4
- Proportion of patients with a pain score < 3 at D11
- Mean % change in bubo size at D4
- Mean % change in bubo size at D11
- Proportion of patients experiencing a serious adverse event on or before D4
- Proportion of patients experiencing a serious adverse event on or before D11
- Proportion of patients experiencing a serious adverse event on or before D21
- Proportion of patients who are fully adherent to the study treatment schedule

##### Pneumonic plague

- Proportion of patients with a therapeutic response at D11. Therapeutic response is defined as follows:
  - ◦ Alive
  - ◦ Resolution of fever (uncorrected axillary temperature < 37.5 °C)
  - ◦ Resolution of tachypnoea (RR < 24 in adults, but age-specific in children)
- Proportion of patients without fever (uncorrected axillary temperature < 37.5 °C) at D4
- Proportion of patients with tachnypnoea resolution (RR < 24 in adults, but age-specific in children) at D4
- Proportion of patients experiencing a serious adverse event on or before D4
- Proportion of patients experiencing a serious adverse event on or before D11
- Proportion of patients experiencing a serious adverse event on or before D21
- Proportion of patients who fully adhere to the study treatment schedule

### Participant timeline {13}

**Fig. 1:**
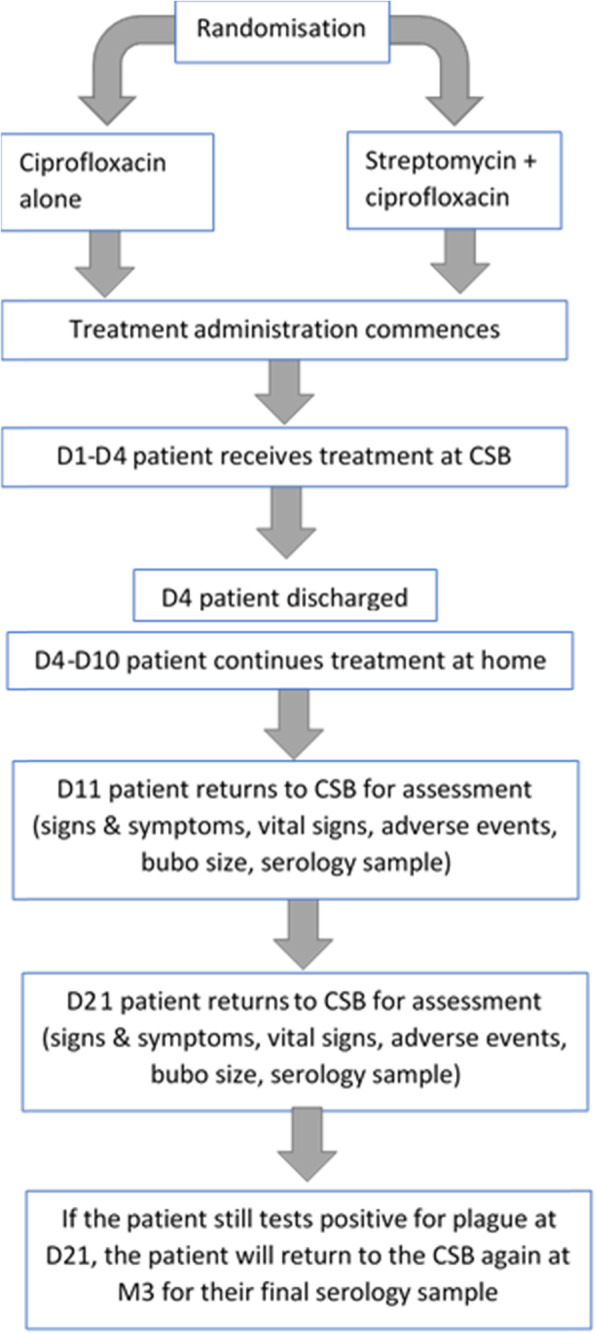
Flow chart demonstrating the participant timeline

The participant timeline is described in the flow chart (Fig. 1).

The schedules of biological procedures, assessments and samples are summarized in the following tables (Tables 1 and 2).

**Table 1:** Schedule of procedures

Variables	Timings (day)												
	1	2	3	4	5	6	7	8	9	10	11	21+/−2	M3
**Informed consent**	X												
**Demographics**	X												
**Medical history**	X												
**Signs and symptoms**	X	X	X	X am							X	X	
**Vital signs**	X	X	X	X							X	X	
**Doses administered at CSB (am and pm)**	XX	XX	XX	X am									
**Doses administered at home (am and pm)**				X pm	XX	XX	XX	XX	XX	XX			
**Adverse events**	X	X	X	X							X	X	
**Bubo size and pain score**	X			X							X	X	

Assessments will be done within 24 h of enrolment, depending on the time of admission

*M3* 3 months after inclusion

**Table 2:** Assessment schedule

Variables	Timing (days)												
	1	2	3	4	5	6	7	8	9	10	11	21+/−2	M3
**RDT Bubo/sputum**	X												
**PCR Bubo/sputum**	X												
**Bubo/sputum culture**	X												
**Plague serology**	X										X	X	X*
**Malaria Ag**	X												

*Only subjects with positive serology on the 21st day will participate in this follow-up

**Fig. 2:**
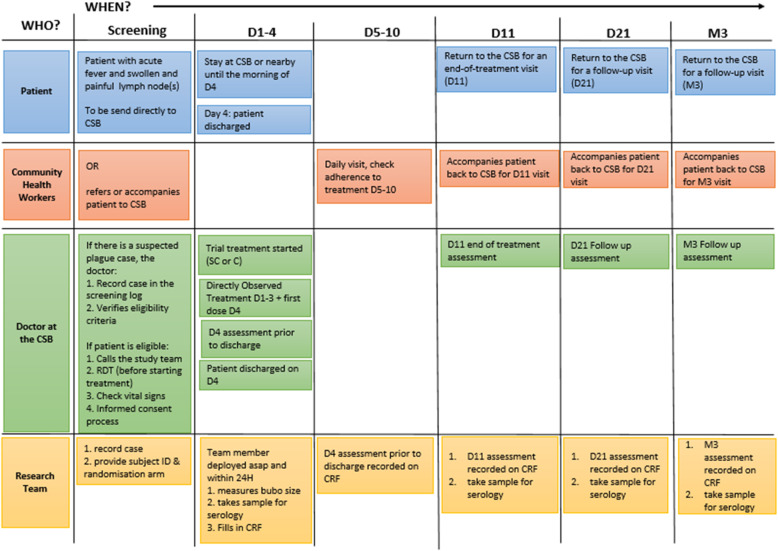
Conduct of the study

The conduct of the study is described in Fig. 2.

The maximum volume of blood to be collected during the entire duration of the study (3 months) for confirmed cases with a positive serology at D21 will be 8 ml for children and 16 ml for adults.

### Sample size {14}

Assuming that 90% of individuals receiving streptomycin plus ciprofloxacin (control treatment) meet the primary endpoint definition of therapeutic response on day 11, 190 confirmed/probable bubonic plague patients (95 per group) would be required to have 90% power to demonstrate the non-inferiority of ciprofloxacin treatment compared to streptomycin plus ciprofloxacin, with a 15% non-inferiority margin and a one-sided confidence interval of 2.5%. This calculation takes into account a 10% loss to follow-up rate by the end of treatment. In total, approximately 600 suspect patients will have to be recruited to obtain a sample size of 190 confirmed/probable patients.

Table 3 shows the total sample sizes (both arms) for confirmed/probable bubonic cases to demonstrate non-inferiority with 90% power, 2.5% one-sided test and assuming 10% loss to follow up, based on treatment success in the streptomycin and ciprofloxacin group and the non-inferiority margin.

**Table 3:** Total sample sizes (both arms) for confirmed/probable bubonic cases

Therapeutic response*	Margin (%), unilateral confidence interval of 2.5%, loss of 10%, power 90%										
	5	6	7	8	9	10	11	12	13	14	15
**0.95**	890	618	454	348	276	224	186	156	134	114	100
**0.90**	1684	1170	858	658	520	424	350	294	250	216	**190**
**0.85**	2384	1656	1216	932	736	596	494	416	354	306	268
**0.80**	2990	2076	1528	1170	926	750	618	520	444	384	334
**0.75**	3506	2434	1790	1370	1084	878	726	610	520	450	392

*Proportion of “therapeutic response” in the streptomycin and ciprofloxacin group

### Recruitment {15}

Sites will be geographically spread throughout pre-identified districts which report a high incidence of plague. Further districts may be included later in the trial in the event that they also report a high incidence of plague.

Whilst it is the responsibility of site medical staff to assess eligibility and enrol patients, the clinical study nurse, who will be based within the district, will review and verify the eligibility of each patient. Community health workers, who will cover a wider geographical area within the districts and reach more remote communities, will also be trained on the protocol and refer suspected cases of plague to the local CSBs.

#### Community engagement and outreach

Outreach teams will disseminate health promotion messages to local health authorities and communities about plague (i.e. signs and symptoms), as well as the background and objectives of the trial.

Community health workers will also raise awareness about going to health centres in the event of suspected plague.

A social science component will address the practical aspects and uptake of the trial that may be affected by the perception of the trial design by both heath care professionals and patients.

## Assignment of interventions: allocation

### Sequence generation {16a}

Bubonic or pneumonic plague patients will be randomized using a computer-generated randomization sequence generated from the master list, stratified by health facility and clinical form of the disease (bubonic or pneumonic plague).

### Concealment mechanism {16b}

The allocation sequence was compiled in STATA in a way that is unpredictable and unreproducible. A copy of the STATA randomization code file used to compile the list can be requested from the trial statistician. The trial statistician will have access and be responsible for the generation and quality assurance of the randomization list. This also includes any changes and/or extensions to the randomization list and date/s when new any treatment groups become effective.

### Implementation {16c}

The trial statistician has generated the allocation sequence.

The doctor in-charge at the CSB will confirm the eligibility of patients with the support of the clinical study nurse. To randomize patients, the doctor in-charge will call the clinical research associate who will randomize the patient on the site’s behalf, informing the site of the treatment allocation over the phone. Contingency plans have been put in place to ensure randomization can take place in the event of internet or mobile signal outage.

## Assignment of interventions: blinding

### Who will be blinded {17a}

Blinding of patients and the trial team to treatment allocation will not be possible due to the different administration routes of the treatments.

### Procedure for unblinding if needed {17b}

Not applicable—this is not a blinded trial.

## Data collection and management

### Plans for assessment and collection of outcomes {18a}

Data will be collected on a paper CRF and then transferred on to a RedCap database for assessment. Automatic validations on RedCap will assess data for completeness and accuracy. A thorough assessment of data accuracy will be performed via regular monitoring activities with a focus on primary endpoint data and safety data, as described in the trial’s monitoring plan.

To validate the methods used to achieve the primary endpoint (% decrease in bubo size), a pilot study and training exercise were conducted ahead of activation in which multiple bubo (real and artificial) measurements were taken using digital callipers (and ultrasound during the pilot). The data were used to assess the concordance of measurements being taken by independent observers. The percentage difference between the sum of the longest and shortest axes of measurements taken by two different clinical study nurse was calculated with an acceptable margin of 25%. The results showed there was less than 25% difference between each measurement.

### Plans to promote participant retention and complete follow-up {18b}

Patients will be provided with a mobile phone upon discharge from the treating centre to optimize retention during follow-up. Community health workers will also assist with reminding patients about their upcoming follow-up visits.

If a patient chooses to withdraw from trial treatment (or the treating clinician believes this is in the best interest of the patient), the patient will continue to receive the standard of care treatment at the discretion of their treating clinician, regardless of the treatment arm to which the patient was randomized. The patient will remain within the trial for the purpose of follow-up, data collection, monitoring and data analysis. Healthcare personnel should explain the importance of allowing existing data to be used, as well as the importance of remaining within the trial for follow-up data collection. If it is not possible to collect further data from the patient at the scheduled trial visits, routine data should be collected via the patients’ medical records.

If a patient withdraws their consent to participate in the trial, data and sample collection will cease and only data up to the point of withdrawal will be used for analysis.

### Data management {19}

All data will be recorded on a paper CRF by clinical study nurses at each study site using a combination of data directly from patients, patient records and laboratory results. The clinical study nurses will then transfer the data from the paper CRF to a RedCap database (version 8.5.17), which will be located centrally at IPM with restricted access. Data queries will be automatically generated by RedCap and transferred to the operational teams in the districts for data quality monitoring as the study progresses. Data monitoring will be conducted on a regular basis as defined in the monitoring plan. The final database will be transferred to Oxford University.

A statistical analysis plan will be prepared prior to the receipt of any data by the trial statistician.

The management, reporting processes and data storage within this trial will comply with the requirements of European Directive (reference 2016/679, “GDPR Directive”) on data protection which is translated into English law by the “Data Protection Act” of May 2018, the Good Clinical Practices of the GCP-ICH, and CIOMS.

### Confidentiality {27}

All the systems used for data management comply with the GCP-ICH and CIOMS recommendations as well as the European Directive (reference 2016/679, “GDPR Directive”) on the protection of personal data, which is translated into English law by the “Data Protection Act” of May 2018.

#### Participant confidentiality

Trial staff will ensure that the participants’ anonymity is maintained. All trial documents will be labelled only with a trial identification number. Trial samples will be labelled only with a trial ID. Routine bubo and sputum samples, which will be analyzed as part of the trial, will be transferred to IPM as per the standard transfer process. The routine samples taken from trial patients will be labelled with a collection label which will not contain a patient trial identifier. A separate letter will be sent to IPM containing anonymised collection information and the patient’s trial ID. This will be used to ensure the results of sample testing from the lab are collected for the trial without compromising the patient’s anonymity.

For the purpose of ensuring data integrity and to facilitate quality assurance, study records will be linked to clinic files, which include the patient’s name and other identifying information. Participants’ names will be recorded confidentially at site in a protected master list at the time of enrolment to allow for their identification at follow-up visits. The master list will not be shared with any other parties involved or not involved in the trial.

All trial data will be stored in a secure database only accessible to trial staff. Only members of the trial team who have completed appropriate data protection training will have access to the password-protected computer where trial data is stored. Study sponsors and health authorities will be given controlled access for the purpose of audit. After the conclusion of the project, data will be removed from the computers and stored in a secure location. Any scientific publications or reports will not identify any patient by name or initials.

### Plans for collection, laboratory evaluation and storage of biological specimens for genetic or molecular analysis in this trial/future use {33}

Aliquots of blood, bubo aspirates and sputum samples will be stored initially at the site and then transported to IPM. Sample custody will be maintained by the investigators, and decisions regarding the use and transfer of samples to the research laboratory will be made by the TOC. Biological research samples will be stored indefinitely, and approval from the sponsor and/or ethics committees, as appropriate, will be sought prior to destruction.

#### Rapid diagnostic test (RDT) for plague at admission

The bubo will be aspirated, and a RDT for plague will be performed on site. This RDT for the detection of the F1 antigen of *Y. pestis* is manufactured by IPM. The test has been used routinely over the past decade and has been validated for bubonic plague. Site staff, clinical research associates and clinical study nurses will be trained on the use and interpretation of the RDT before the start of the study and during annual retraining.

#### At the central plague laboratory (CPL) level

The remaining bubo aspirate will be stored in Cary Blair transport medium, packed according to the required safety level using the materials already provided (triple packaging), then sent to the CPL at IPM for a second RDT analysis as well as for molecular and bacteriological diagnosis. Molecular diagnosis will first be performed by real-time PCR (qPCR) targeting 2 fragments of the *pla* and *caf-1* genes of *Y. pestis*, and only samples with inconclusive results will be confirmed by conventional PCR (cPCR) targeting 3 genes *pla*, *caf-1* and inv1100. Bacteriology will be done with direct culture and amplification in mice followed by biochemical identification of the suspected strain by API 20E and by bacteriophage lysis test, then antibiotic susceptibility testing of each *Y. pestis* isolated strain according to the Kirby Bauer method interpreted according to the CLSI reference.

Sputum samples will not be tested on site with RDT but will be sent directly to the CPL. Biological results will be sent back to the physician responsible for the patient.

#### Malaria

A malaria RDT will be performed on all patients at admission.

#### Serology

A 4-ml venous blood sample will be taken at admission (D1), D11 and D21 for plague serology (for children, a 2-ml venous blood sample will be taken at these time points). In addition, for subjects with positive serology at D21, serological follow-up will be carried out at 3 months (M3) after their inclusion. Two types of serological techniques will be performed: serology by immuno-enzymatic assay method (ELISA, anti-F1 antibodies, IgG) which is validated and will be performed at the CPL, and serological analysis by xMAP-Luminex multiplex technique (Magpix) to simultaneously detect antibodies (IgM and IgG) directed against several *Y. pestis* antigens will be performed at the immunology of infectious diseases unit (IMI) at IPM. A serological test (on magnetic beads, XMAP-Luminex) to detect and quantify the F1 circulating antigen of *Y. pestis* will also be performed. Only anti-F1 IgG ELISA technique will be considered for case classification.

## Statistical methods

### Statistical methods for primary and secondary outcomes {20a}

#### Primary endpoint analysis

The final classification of cases will take into account all clinical and biological data in accordance with WHO recommendations adapted in 2017 with the introduction of qPCR as a diagnostic tool [3, 11].

The data will be analyzed using four analysis populations: (i) intention to treat (ITT), (ii) intention to treat infected (ITTI), (iii) per-protocol (PP) and (iv) per-protocol infected (PPI). Intention to treat infected (ITTI) will be the primary analysis population. An infected patient is a confirmed or probable case. A case is probable if the RDT or qPCR or serology (anti-F1 IgG ELISA) is positive but without evidence of seroconversion or a fourfold increase in antibody titre. A case is confirmed if the RDT and qPCR are positive or if the culture is positive or if there has been a seroconversion or a fourfold increase in antibody titre on two separate serological samples (either between D1 and D11, between D11 and D21 or between D1 and D21).

The criteria for exclusion from the per-protocol analysis population will be specified in advance in the statistical analysis plan (SAP). The proportion of patients who meet the pre-established definition of therapeutic response in the streptomycin plus ciprofloxacin group will be subtracted from the proportion of patients who meet the pre-established definition of therapeutic response in the ciprofloxacin group. A positive difference in proportions would imply a higher response in patients treated with ciprofloxacin, compared to streptomycin plus ciprofloxacin, and a negative difference would imply a lower response in patients treated with ciprofloxacin. If the lower limit of the one-sided 2.5% confidence interval (CI) around the difference in proportions is not less than − 0.15, ciprofloxacin will be considered non-inferior to streptomycin plus ciprofloxacin.

#### Analysis of secondary endpoints

Descriptive summaries of the data will be provided by randomization arm for the pre-specified secondary outcomes. The data will be summarized by randomization arm using the mean (95% Cl) and median (interval) for continuous or discrete data and raw data with percentages and 95% CIs for binary variables. The average time to resolution of fever and pain at the bubo site will also be summarized.

### Interim analyses {21b}

No formal interim analysis is planned. It is unlikely that the trial can be stopped prematurely, as a smaller sample will not provide the precision required to demonstrate non-inferiority within the pre-specified margin.

### Methods for additional analyses (e.g. subgroup analyses) {20b}

No additional analyses are planned.

### Methods in analysis to handle protocol non-adherence and any statistical methods to handle missing data {20c}

#### Missing data handling: primary endpoint

If a patient has had a resolution of fever and shows a ≥ 25% reduction in bubo size on or after day 4, but is lost for follow-up before day 11, he or she will be considered to have had a therapeutic response.

#### Protocol deviations

Any deviations from the protocol will be fully documented on a protocol deviation form that will be sent to the sponsor and stored in the trial master file and on site in the study site file.

### Plans to give access to the full protocol, participant-level data and statistical code {31c}

There are currently no plans to grant public access to the full protocol, participant-level dataset or the statistical code.

## Oversight and monitoring

### Composition of the coordinating centre and trial steering committee {5d}

IPM will jointly coordinate the trial with the sponsor, the University of Oxford.

As well as a central trial coordination team to coordinate the day-to-day activities of the trial, IPM will deploy one clinical research associate and two clinical study nurses to each district who will assist the sites with the recruitment, data collection and conduct monitoring activities. IPM will also be primarily responsible for data management but supported by the University of Oxford.

The University of Oxford will coordinate the sponsor activities, coordinate the trial committees and provide day-to-day management and oversight of the trial.

The TOC is coordinated by the University of Oxford and is composed of members from each of the study partners to review the operational management of the trial and discuss any urgent matters related to trial conduct as and when they arise. The group meets on a weekly basis to discuss study progress.

The Trial Steering Committee provides overall supervision for the trial on behalf of the trial sponsor and to ensure that the study/trial is conducted according to the guidelines for Good Clinical Practice (GCP) and all relevant regulations and local policies.

A Data Safety Monitoring Board has also been established to maintain oversight of patient safety and data integrity within the trial.

### Composition of the data monitoring committee, its role and reporting structure {21a}

An independent Data Safety Monitoring Board (DSMB) has been established to safeguard the interests of trial participants, to assess the safety of the interventions during the trial and to assist and advise the principal investigator (PI) so as to protect the validity and credibility of the trial.

The DSMB will meet remotely (1) upon the opening of the trial, (2) at the end of each plague season and (3) at the end of the trial.

DSMB members:

- Paul Mead, Chief of the Bacterial Diseases Branch (BDB), Division of Vector-Borne Diseases (DVBD), National Center for Emerging and Zoonotic Infectious Diseases (NCEZID), Centres for Disease Control and Prevention
- Professor David Lalloo, Director Liverpool School of Tropical Medicine and Professor of Tropical Medicine, Liverpool School of Tropical Medicine, Pembroke Place Liverpool, L3 5QA, UK
- Professor Julie A Simpson, Head of Biostatistics Unit/Deputy Head of School, Centre for Epidemiology and Biostatistics, Melbourne School of Population and Global Health, The University of Melbourne, Victoria 3010, Australia

### Adverse event reporting and harms {22}

Streptomycin and ciprofloxacin are approved drugs with well-established safety profiles. Serious adverse events/serious adverse reactions (SAEs/SARs) and suspected unexpected serious adverse reactions (SUSARs) will be reported in accordance with the international guidelines and applicable national legislation.

All adverse events (AEs) observed or reported by the patient should be recorded on the AE form within the CRF.

In the event of an adverse event or reaction, the investigator is responsible for providing an appropriate medical response, providing a causality assessment and monitoring the progress of the AE until it has resolved. In the event that an adverse event is deemed serious, according to the international definition (ICH), it should be notified immediately to IPM via the SAE report form within 48 h of the site becoming aware of the event. Newly arising SAEs must be reported from the point of consent until D21. All SAEs must be followed up by the site until resolution (including when resolution occurs after D21).

The following events, which are either classified as treatment evaluation criteria or inclusion criteria, are exempt from reporting as an SAE:

- Respiratory symptoms (cough, tachypnea, haemotysis)
- Intestinal symptoms (diarrhoea, vomiting)
- Fever
- Bubo
- Pain at bubo’s site

In addition, the following circumstances should not be notified to the sponsor:

- Routine examination
- Medical/surgical/hospital procedure planned before inclusion
- A condition present before inclusion or discovered at inclusion (e.g. malaria discovered at inclusion and requiring hospitalization)

All SAEs/SARs reported to IPM will be notified to the sponsor within one business day and will be reviewed and evaluated by two clinical reviewers. If the reviewers have concerns about the SAEs submitted, an emergency meeting of the DSMB and the TOC will be convened to discuss the management of the SAE and the potential impact on the trial.

All SUSARs will be reported to the Centre National de Pharmacovigilance in Madagascar within 7 days for fatal or life-threatening reports and within 15 days for other serious, unexpected SARs for all initial reports.

### Frequency and plans for auditing trial conduct {23}

None.

### Plans for communicating important protocol amendments to relevant parties (e.g. trial participants, ethical committees) {25}

All protocol amendments will be discussed by the TOC which includes members from each of the study partners ahead of submission to the respective ethics committees. It is the responsibility of each institution to obtain their own ethical approvals. Only once all ethical approvals have been obtained will the amendment be implemented. It will be the responsibility of IPM to disseminate the changes to the protocol to participating sites.

### Dissemination plans {31a}

The principal publication of the study will be in the name of the investigators with full credit assigned to all collaborating investigators, research coordinators and institutions. Oral or written communications will acknowledge all trial stakeholders, either in the list of authors or in the acknowledgements, depending on their respective contributions to the trial and the presentations made. A drafting committee will be formed at the time of data analysis. The order of authors at publication will be equally contributed, by the importance of contribution or in alphabetical order. Results from the trial will be published in open-access journals, and the data will be available for sharing.

## Discussion

Conducting clinical trials of plague is operationally challenging, mostly because cases occur in rural places with difficult access to health facilities, especially because cases occur mostly during the rainy season, and are scattered over a vast territory. Communication and network coverage may be erratic, and security issues may also occur. This requires adequately staffed, well-trained study teams to be deployed over a number of peripheral health facilities, with logistical challenges and inherent high costs. This, plus the fact that drugs are registered for plague (based on the ‘animal rule’) and that national and international guidelines exist (based on empirical regimens), along with the general lack of funding options for plague, has essentially discouraged the conduct of treatment trials.

## Trial status

Protocol version number: 2.9

Protocol date: 27 September 2019

Recruitment start date: planned February 2020

Planned recruitment end date: March 2023

## Supplementary information

**Additional file 1.** : Patient information sheet. Informed consent form (ADULTS). Informed consent form (CHILDREN AND PROXY).

**Additional file 2.** Case Report Form (CRF).

## Abbreviations

AE: Adverse event
C: Celsius
caf-1 gene: Chromatin assembly factor-1
CI: Confidence interval
CIOMS: Council of International Organizations of Medical Sciences
CLSI: Clinical & Laboratory Standards Institute
CPL: Clinical plague laboratory
CRF: Case report form
CSB: Centre de Santé de Base
D: Day
DFID: Department for International Development
DSMB: Data and Safety Monitoring Board
ELISA: Enzyme-linked immunosorbent assay
F1: Fraction 1 antigen
FDA: Food and Administration
GCP: Good Clinical Practice
GDPR: General Data Protection Regulation
IgG: Immunoglobulin G
IgM: Immunoglobulin M
Inv1100 gene: Chromosomal invasin gene
IPM: Institute Pasteur de Madagascar
ITT: Intention to treat
ITTI: Intention to treat infected
M3: Month 3
PI: Principal investigator
PP: Per-protocol
PPI: Per protocol infected
qPCR: Quantitative polymerase chain reaction
RCT: Randomized clinical trial
RDT: Rapid diagnostic test
RR: Respiratory rate
SAE: Serious adverse event
SAP: Statistical analysis plan
TOC: Trial Operations Committee
WHO: World Health Organization
WT: Wellcome Trust
