# Supplementary material for: An open-label, randomized, non-inferiority trial of the efficacy and safety of ciprofloxacin versus streptomycin + ciprofloxacin in the treatment of bubonic plague (IMASOY): study protocol for a randomized control trial
Source: Trials. 2020 Aug 17;21:722. doi: 10.1186/s13063-020-04642-2 (PMC7429934; doi:10.1186/s13063-020-04642-2)
Supplement: Supplementary file 1 — Additional file 1. : Patient information sheet. Informed consent form (ADULTS). Informed consent form (CHILDREN AND PROXY). [file 13063_2020_4642_MOESM1_ESM.docx]

***Patient Information Sheet***

**IMASOY: An open-label, randomised, non-inferiority trial of the efficacy and safety of ciprofloxacin versus ciprofloxacin and streptomycin for the treatment of bubonic plague**

**Version 2.3**

**21-Oct-2019**

OxTREC Version 45-18

**Principal Investigator Madagascar** : Professor Mamy Jean de Dieu Randria

Contact details: 034 14 200 30, rmamyjeandedieu@yahoo.fr

**Principal Investigator United Kingdom:** Professor Peter Horby

**Contact details:** 07990560237, Peter.horby@ndm.ox.ac.uk

**Co-investigators**: Dr. Laurence Baril, Institut Pasteur de Madagascar, Dr. Tansy Edwards, London School of Tropical Medicine and Hygiene, Dr Piero Olliaro, Dr. Mihaja Raberahona, University Hospital Joseph Raseta Befelatanana, Dr. Minoarisoa Rajerison, Institut Pasteur de Madagascar, Dr. Rindra Randremanana, Institut Pasteur de Madagascar, Dr. Alex Salam, University of Oxford.

**Research location**: [Add name and address of health centre]

**Background**

You and other patients with plague at this centre are invited to take part in a research study to test the efficacy of a treatment for plague. The study is being done by University Hospital Joseph Raseta Befelatanana Antananarivo, Institut Pasteur de Madagascar and the University of Oxford (by a team led by Professor Mammy Randria, and Professor Peter Horby). Please read this information sheet carefully, or someone can read it to you. You will be given a copy of this form if you decide to take part in the study and a copy to keep when you leave the treatment centre. Before you decide if you would like to take part, it is important for you to understand why this study is being done and what will happen if you take part. Please ask questions if there is anything you do not understand or would like to know more about.

**Why is the study being conducted?**

Plague is an infection caused by bacteria. The main types of plague are bubonic plague, which causes painful and swollen glands and fever, and pneumonic plague, which causes cough and fever. It is important to treat plague early and effectively to give patients the best chance of recovery. There are different types of antibiotics that can be used to treat plague. In Madagascar, the current treatment for plague in adults involves two antibiotics for bubonic plague, streptomycin for 3 days followed by ciprofloxacin for 7 days, and two antibiotics for pneumonic plague, both streptomycin and ciprofloxacin for 5 days then ciprofloxacin alone for a further 5 days. Streptomycin has been used for many years in Madagascar and other countries. Streptomycin however is given by an injection into the muscle twice a day, which can be painful. It can also result in serious side effects in a proportion of patients, the main ones being kidney impairment and hearing and balance impairment. In addition, in a few years the manufacture of streptomycin will be stopped and it will be difficult to buy the drug. Ciprofloxacin is an antibiotic that is used for many different infections. It has been used in some plague patients, but not many, and there has been no research into how well it works in humans. Because of the side effects associated with streptomycin and the fact that it is given by injection into a muscle, we want to test whether ciprofloxacin given on its own, is as effective as streptomycin in combination with ciprofloxacin for the treatment of plague. Data from experiments in laboratories, in animals and from its use in some humans suggests that ciprofloxacin is a very good treatment for plague when used on its own. However, to date there have been no clinical trials of ciprofloxacin for the treatment of plague. As a result, ciprofloxacin has not been used routinely for plague patients and has not been used as the first choice of treatment for plague. Ciprofloxacin does however have several potential advantages over streptomycin and other potential treatments for plague. It can be given orally or through a vein, it is safer in pregnancy, and it does not require kidney or drug level monitoring. This study therefore aims to test whether ciprofloxacin alone is as effective as ciprofloxacin in combination with streptomycin for the treatment of plague. Demonstrating this would enable doctors and nurses to routinely use ciprofloxacin alone for the treatment of plague.

**What will happen if you take part in the study?**

If you decide to take part in this study you will be randomly selected to receive either streptomycin for 3 days followed by ciprofloxacin for 7 days, or ciprofloxacin alone for 10 days. The staff treating you and the research staff won’t know which treatment you will receive in advance, and they will have no influence over which treatment you will randomly receive. The random selection process will have been decided in advance by a computer program. Patients are randomly allocated to a specific treatment so that both treatment groups are as similar as possible in terms of things such as age, pregnancy status and health status. This is very important to ensure that the patient characteristics are the same in each treatment group.

Before you receive either treatment, you will have some pus taken from any swollen glands. This will involve inserting a small needle into the gland and withdrawing the pus. This is routinely done for all patients who have bubonic plague in Madagascar, and would be done whether or not you took part in this study. The pus will then be tested for the presence of plague. If you are coughing, some of your sputum will be collected and tested for plague. Again, this would be done as standard whether or not you took part in the study. You will also have some blood taken from a vein. This blood will be tested for antibodies against plague. Antibodies are proteins made by your immune system that help fight plague. The total volume of blood to be collected will be 16 ml for adults, which is equivalent to about 3 teaspoons and 8 ml for children, which is equivalent to about 1.5 teaspoons.

For subjects with a positive blood test at D21, the total volume of blood to be collected will be higher, and they will have to repeat the blood test at the 3rd month, after their hospitalization to see the changes in their body's immune system.

Photographs can be taken of potential buboes during bubo measurements throughout the study on day 1, day 4, day 11 and day 21 of the study.

If you are randomly selected to receive streptomycin and ciprofloxacin, you will be given streptomycin by an injection into one of your muscles for 3 days. After 3 days, you will then be given ciprofloxacin given in tablet form orally for 7 days. If you are randomly selected to receive ciprofloxacin alone, you will receive this in tablet form orally for 10 days. If you are unable to take oral tablets, you will be given ciprofloxacin by injection into the vein. If your doctor or nurse feels that an injection of ciprofloxacin would be better for you, you will be given ciprofloxacin by injection into the vein. Once you can take ciprofloxacin orally, the injection will be stopped and you will be given ciprofloxacin tablets.

You will be seen by a member of the research team every day whilst you are being treated for plague. After you have finished treatment, you will be seen by the research team once, 21 days after you received your first dose of treatment for plague. For subjects with positive blood results, they will be seen in addition to the 3rd month, after their stay in the health facilities. This will be either at the health centre or at your home.

If you don’t want to be in this study, you don’t have to participate. Remember, being in this study is up to you and no one will be upset if you don’t want to participate or even if you change your mind later and want to stop. You can ask any questions that you have about the study. If you have a question later that you didn’t think of now, you can ask a member of the research team next time.

The study team may also stop your involvement in the study at any time if they feel this is in your best interests. However, because there are minimal risks involved in your participation in the study, this is unlikely.

**Are there any risks or disadvantages to you when taking part?**

If you are taking part in the study, there are a few small risks or disadvantages. Inserting a cannula to give you an injection through the vein if you need it may cause mild soreness, bleeding or bruising where the needle went in. Very occasionally inserting a cannula can lead to infection, but we will only keep the cannula for a few days, which makes the risk very small. There is also an inconvenience in taking extra blood samples. Streptomycin can cause kidney impairment or hearing loss, and the muscle injections can be painful. However, if you were not taking part in this study you would receive this as part of the standard treatment for plague in Madagascar anyway. Ciprofloxacin can very rarely cause inflammation of tendons or psychiatric symptoms. However, if you were not taking part in this study you would receive this as part of the standard treatment for plague in Madagascar anyway.

**Are there any benefits to me taking part?**

This study may not definitely help you directly. You will not be paid for taking part in this study. If you are returning each day to the treatment centre to receive your treatment for plague, the cost of your transport will be refunded by the research team.

**What happens if I do not want to take part?**

You do not have to take part in the study. If you decide to be in the study, you can stop at any time without giving a reason. Staff at the treatment centre will give you the best available care whether you take part or not. Only the information collected before stopping the study will be used. As plague treatment is very important to you and your community whether or not you participate in the trial, if you withdraw from the trial before the end of your treatment, the medical staff at the centre will continue your treatment in accordance with the national plague management protocol in Madagascar.

**What will happen to my information?**

All information about you will be kept confidential by those working on this study. Your name will not be used on your blood tests, data or in any reports about this study. All the study data will be entered into a database that will be password protected, and only members of the research team will be able to look at this data. After completion of the study and publication of the results, your anonymised data will be stored in a database that other researchers will have access to. Importantly, there will nothing in this data that can be used to identify you.This database will be located at Oxford University. Your data will be stored for a minimum of 5 years after the results of the study have been published.

**What will happen with my blood and body fluid samples?**

Your blood samples will be tested for the response of your body to plague infection and treatment. Any leftover blood or pus or sputum will be stored at Institut Pasteur de Madagascar in case we want to do more tests in the future. Such tests may include, for example, further tests of your immune system or further tests on the plague bacteria. None of your samples will have any information that can identify you. Your samples will be stored for a minimum of 5 years after the results of the study have been published.

**Who has allowed this study to take place?**

This study has been approved by the ethics committees of: Madagascar National Ethics Committee (CERBM) and the University of Oxford (OxTREC). These committees have looked carefully at this study and agreed that the research is important, it will be conducted properly and the safety and rights of patients who take part have been respected.

**Data protection**

Oxford University is responsible for ensuring the safe and proper use of any personal information you provide, solely for research purposes.

**What if you have any questions?**

You are free to ask me, or another member of the study team, any questions about this research. We will be happy to answer any questions or concerns you might have anytime. You are also welcome to talk to family and friends, or ask someone independent, before you agree to take part. If you have questions after you leave the treatment centre, you can call the trial staff. They can also give you other numbers for information about your rights as a participant.

You may also contact the Madagascar Biomedical Research Ethics Committee (020 22 365 22) or the Principal Investigator of the trial (034 14 200 30) if you believe that your rights as a research participant have not been respected or if you suffer an injury as a result of your participation in this trial.

***Informed Consent Form (ADULTS)***

**IMASOY: An open-label, randomised, non-inferiority trial of the efficacy and safety of ciprofloxacin versus ciprofloxacin and streptomycin for the treatment of bubonic plague**

**It is your choice if you want to participate or not. Read the following statements and if you agree, please initial the box next to the statements:**

- All of the information above is clear, I understand the risks and benefits of the study and that any questions I may have asked about this study were answered appropriately.


- I agree to be randomly selected to receive either ciprofloxacin alone OR streptomycin in combination with ciprofloxacin


- Any leftover blood can be stored and used for plague research in the future.


- I understand I can end my participation at any time and I will still receive the best available care at the treatment centre.


- I agree to take part in this trial



Time and date that information was discussed. Time ___:___ Date ___/___/______

Patient name: __________________________________ Patient Identification Number: ___________________

Time and date of signature Time ____:____ Date ___/___/_____

| Patient signature:  x_____________________________________________________ |
| --- |

| I have followed the study procedures to obtain informed consent. The patient (or their representative) has freely agreed to participate. Study staff signature:  x____________________________ | Study staff name:  x______________________________ |
| --- | --- |

If the person giving consent cannot read the consent form by themselves, a witness should be present to hear this form read accurately to the patient and ensure that the study is explained to them such that they can understand. The person signing below confirms that informed consent was freely given by the patient.

| Witness signature:  x___________________________ | Witness name:  x____________________________________ |
| --- | --- |

***Informed consent form (CHILDREN AND PROXY)***

**IMASOY: An open-label, randomised, non-inferiority trial of the efficacy and safety of ciprofloxacin versus ciprofloxacin and streptomycin for the treatment of bubonic plague**

**It is up to you to choose whether or not you want your friend, relative or representative to participate in this trial. Read the following statements and if you agree, please initial the box next to the statements:**

- All the above information is clear, I understand the risks and benefits of the trial and any questions I may have asked about this trial have been appropriately answered.


- I agree that the participant may be randomly selected to receive ciprofloxacin alone, OR streptomycin (adults) or gentamicin in combination with ciprofloxacin.


- All remains of blood and other body fluids can be stored and used for plague research in the future.


- I understand that I can terminate the member's participation at any time and that he or she will always receive the best care available at the treatment centre.


- I agree for my friend, relative or representative to take part in this trial

The time and date on which this information was discussed. Time ___:___ Date ___/___/_________

Patient __________________________________ 's name : ______________________

Patient ___________________identification number: ____________

Time and date of signature Time ____:_______ Date ___/___/________

| Signature of the person giving consent:  __________________________ x | Name of the person giving consent:  _______________________ x | Relationship to the patient:  _________________________ x |
| --- | --- | --- |

| I followed the trial procedures to obtain informed consent. The patient (or his or her friend, relative or representative) has freely agreed to participate. Signature of the trial staff:  ____________________________ x | Name of trial staff:  ______________________________ x |
| --- | --- |

If the person giving consent cannot read the form by himself or herself, a witness should be present to hear the form accurately read to the patient and ensure that the trial is explained to him or her so that he or she can understand. The person signing below confirms that informed consent has been freely given by the patient.

| Witness' signature:  ___________________________ x | Name of the witness:  ____________________________________ x |
| --- | --- |
