# Supplementary material for: An open-label, randomized, non-inferiority trial of the efficacy and safety of ciprofloxacin versus streptomycin + ciprofloxacin in the treatment of bubonic plague (IMASOY): study protocol for a randomized control trial
Source: Trials. 2020 Aug 17;21:722. doi: 10.1186/s13063-020-04642-2 (PMC7429934; doi:10.1186/s13063-020-04642-2)
Supplement: Supplementary file 2 — Additional file 2. Case Report Form (CRF). [file 13063_2020_4642_MOESM2_ESM.docx]

| **INCLUSION DU PATIENT** |
| --- |

1. **CRITERES D’INCLUSION**

| **PESTE PULMONAIRE / PESTE MIXTE** ☐ OUI ☐ NON | |
| --- | --- |
| Patients de tout âge et sexe ET | ☐ OUI ☐ NON |
| Apparition récente (< 7 jours) de fièvre (température axillaire non corrigée >37,5°C) ou notion de fièvre ET | ☐ OUI ☐ NON |
| Toux ET | ☐ OUI ☐ NON |
| Tachypnée (fréquence respiratoire > 24 cycles/min chez les adultes et spécifique à l'âge chez les enfants) ET | ☐ OUI ☐ NON |
| Lien épidémiologique avec un cas confirmé ou probable de peste pneumonique primaire ou secondaire dans les 7 jours avant l'apparition des symptômes. | ☐ OUI ☐ NON |
| OU le patient a développé une toux et une dyspnée après l’apparition du bubon | ☐ OUI ☐ NON |

| **PESTE BUBONIQUE** ☐ OUI ☐ NON | |
| --- | --- |
| Patients de tout âge et sexe ET | ☐ OUI ☐ NON |
| Apparition récente (< 10 jours) de fièvre (température axillaire non corrigée >37,5°C) ou notion de fièvre) ET | ☐ OUI ☐ NON |
| Un ou plusieurs bubons (gonflement des ganglions lymphatiques) ET | ☐ OUI ☐ NON |
| Résidence ou voyage dans une zone d'endémie de peste à Madagascar dans les 14 jours avant l'apparition des symptômes. | ☐ OUI ☐ NON |
| Patients identifiés comme cliniquement suspects de peste par le personnel de santé (médecins ou para-médicaux) | ☐ OUI ☐ NON |

1. **CRITERES DE NON INCLUSION**

| Si un des critères est coché **OUI**, le sujet **ne peut pas** être inclus | |
| --- | --- |
| Allergie connue aux aminoglycosides ou aux fluoroquinolones. | ☐ OUI ☐ NON |
| Tendinite | ☐ OUI ☐ NON |
| Myasthénie grave | ☐ OUI ☐ NON |
| Utilisation de théophylline ou de warfarine | ☐ OUI ☐ NON |
| Femme enceinte | ☐ OUI ☐ NON ☐ NON APPLICABLE |
| Déjà traités pour une peste bubonique ou pneumonique au cours des 3 mois précédents | ☐ OUI ☐ NON |

1. **INFORMATION CONCERNANT LE CONSENTEMENT/RANDOMISATION**

| **CONSENTEMENT** | |
| --- | --- |
| **Consentement** | ☐OUI ☐NON |
| **Si consentement signé, photographie du consentement** | ☐OUI ☐NON |
| **Nom du site** |  |
| **Date de consentement** | [__][__]/ [__] [__] / [__] [__] [__] [__] |
| **Heure du consentement** | [__][__]/ [__] [__] |
| **Nom de la personne prenant le consentement** |  |
| **CONCLUSION SUR L’INCLUSION** | |
| **Le sujet est inclus dans l’étude ?** | ☐ OUI ☐ NON |
| **Si NON INCLUS, merci d’indiquer la raison** | ☐ PATIENT ELIGIBLE ET NON INCLUS  (*répond aux critères d'inclusion mais au moins un critère d'exclusion est coché)*  ☐REFUS DU PATIENT  ☐NON ELIGIBLE  ☐AUTRES, précisez: _________________________________________________________________________________________________________________________________ |
| **Type de peste suspectée** | ☐ Bubonique ☐ pulmonaire/mixte |
| **Nom du site** |  |
| **Date de consultation/admission** | [__][__]/ [__] [__] / [__] [__] [__] [__] |
| **Heure de consultation/admission** | [__] [__]/ [__] [__] |
| **RANDOMISATION** | |
| **Date de rand omisation** | [__][__]/ [__] [__] / [__] [__] [__] [__] |
| **Heure de randomisation** | [__] [__]/ [__] [__] |
| **Bras de randomisation** | ☐ STREPTOMYCINE ET CIPROFLOXACINE  ☐ CIPROFLOXACINE |

| **VISITE V1** |
| --- |

1. **VISITE V1**

| **Date prévisionnelle de la visite** | [__][__]/ [__] [__] / [__] [__] [__] [__] |
| --- | --- |
| **Jour de la visite** | 🞎J1 🞎J2 🞎J3 🞎J4  🞎J5 🞎J6 🞎J7 🞎J8 🞎J9 🞎J10  🞎J11 🞎J21  🞎M3 |
| **Lieu de la visite** | 🞎 Sur site 🞎 A domicile |
| **La visite a –t-elle pu être réalisée ?** | 🞎OUI 🞎NON |
| **Patient vu le jour de la visite, par :** | 🞎 Médecin / Responsable du site  🞎 TEC  🞎 Agent communautaire |
| **Date effective de la visite** | [__][__]/ [__] [__] / [__] [__] [__] [__] |
| **Déclaration évènement indésirable** | 🞎OUI, *si « oui » compléter le formulaire EI*  🞎NON |
| **Si la visite n’a pas pu être réalisée, précisez la raison :** | 🞎 Arrêt prématuré *(Compléter le formulaire « SORTIE prématurée/Fin d’étude »)*  🞎 AUTRE, précisez la raison :**_____________________________________________________** |

1. **DONNEES DEMOGRAPHIQUES**

| **Sexe** | ☐Masculin ☐Féminin |
| --- | --- |
| **Âge**  *(Remplir 00 dans « ans » si individu < à 24 mois ;*  *remplir 00 dans « mois » si l’individu à un âge exact, exemple : 12 ans)* | [___] [___] ans  [___] [___] mois |
| **Poids** | [___] [___] , [___][___][___] kg |

1. **COMORBIDITÉS** *(préexistantes AVANT L’ADMISSION et qui continuent à être un problème)*  **ET TRAITEMENTS ASSOCIES**

| **Pathologie cardiaque** | 🞎OUI 🞎NON 🞎NE SAIT PAS | Diurétiques 🞎OUI 🞎NON 🞎NE SAIT PAS |
| --- | --- | --- |
| **BPCO / asthme** | 🞎OUI 🞎NON 🞎NE SAIT PAS | Stéroïdes inhalés 🞎OUI 🞎NON 🞎NE SAIT PAS |
| **Diabète** | 🞎OUI 🞎NON 🞎NE SAIT PAS | Hypoglycémiants oraux 🞎OUI 🞎NON 🞎NE SAIT PAS  Insuline 🞎OUI 🞎NON 🞎NE SAIT PAS |
| **Insuffisance rénale chronique** | 🞎OUI 🞎NON 🞎NE SAIT PAS | Dialyse 🞎OUI 🞎NON 🞎NE SAIT PAS |
| **HIV** | 🞎OUI 🞎NON 🞎NE SAIT PAS | Antirétroviraux 🞎OUI 🞎NON 🞎NE SAIT PAS |
| **Autres, précisez :** | | |
| **Comorbidité 1** |  | |
| **Comorbidité 2** |  | |
| **Comorbidité 3** |  | |
| **Comorbidité 4** |  | |
| **Comorbidité 5** |  | |

1. **SOINS AVANT INCLUSION**

| **Traitement de cet épisode de la maladie au cours de 7 Jours précèdent l’admission** : 🞎OUI 🞎NON 🞎INCONNU | |
| --- | --- |
| **Nom de l’antibiotique 1 :** | **__________________________________** |
| **Date de début :** | [__][__]/ [__] [__] / [__] [__] [__] [__] |
| **Nombre de jours pris :** | [__] [__] |
| **Nom de l’antibiotique 2 :** | **__________________________________** |
| **Date de début :** | [__][__]/ [__] [__] / [__] [__] [__] [__] |
| **Nombre de jours pris :** | [__] [__] |
| **Nom de l’antibiotique 3 :** | **__________________________________** |
| **Date de début :** | [__][__]/ [__] [__] / [__] [__] [__] [__] |
| **Nombre de jours pris :** | [__] [__] |
| **La prophylaxie antibiotique pris avant cette admission :** 🞎OUI 🞎NON 🞎INCONNU | |
| **Nom de l’antibiotique 1 :** | **__________________________________** |
| **Date de début :** | [__][__]/ [__] [__] / [__] [__] [__] [__] |
| **Nombre de jours pris :** | [__] [__] |
| **Nom de l’antibiotique 2 :** | **__________________________________** |
| **Date de début :** | [__][__]/ [__] [__] / [__] [__] [__] [__] |
| **Nombre de jours pris :** | [__] [__] |
| **Nom de l’antibiotique 3 :** | **__________________________________** |
| **Date de début :** | [__][__]/ [__] [__] / [__] [__] [__] [__] |
| **Nombre de jours pris :** | [__] [__] |

1. **SYMPTOMES / SIGNES CLINIQUES – V1**

| **EXAMEN CLINIQUE** | | |
| --- | --- | --- |
| **Date d’évaluation :** [__][__]/ [__] [__] / [__] [__] [__] [__] **Heure de l'évaluation :** [__] [__] / [__] [__]  **Date d'apparition du premier symptôme :** [__][__]/ [__] [__] / [__] [__] [__] [__] | | |
| **Fièvre** | ☐OUI ☐NON ☐Inconnu Depuis combien de temps en jours [__] [__] | |
| **Toux** | ☐OUI ☐NON ☐Inconnu Depuis combien de temps en jours [__] [__] | |
| **Crachats purulents** | ☐OUI ☐NON ☐Inconnu Depuis combien de temps en jours [__] [__] | |
| **Crachats sanglants** | ☐OUI ☐NON ☐Inconnu Depuis combien de temps en jours [__] [__] | |
| **Hémoptysie franche** | ☐OUI ☐NON ☐Inconnu Depuis combien de temps en jours [__] [__] | |
| **Dyspnée** | ☐OUI ☐NON ☐Inconnu Depuis combien de temps en jours [__] [__] | |
| **Douleur thoracique** | ☐OUI ☐NON ☐Inconnu Depuis combien de temps en jours [__] [__] | |
| **Bubon(s)** | ☐OUI Depuis combien de temps en jours [__] [__] Nombre de bubons  [__][__]  ☐NON ☐Inconnu | |
| **Site du Bubon(s)** | Axillaire | ☐OUI ☐NON  ☐ Gauche ☐ Droite  ☐Visible et mesurable ☐Palpable et non mesurable  ☐Ferme ou ☐Souple  Rougeur ☐OUI ☐NON  Œdème des tissus ☐OUI ☐NON  Suppuration ☐OUI ☐NON  Score de douleur ____  Taille  (mm) : Grand Axe [___] [___],[___] [___] Petit Axe [___] [___],[___] [___] |
|  | Cervical | ☐OUI ☐NON  ☐ Gauche ☐ Droite  ☐Visible et mesurable ☐Palpable et non mesurable  ☐Ferme ou ☐Souple  Rougeur ☐OUI ☐NON  Œdème des tissus ☐OUI ☐NON  Suppuration ☐OUI ☐NON  Score de douleur ____  Taille  (mm) : Grand Axe [___] [___],[___] [___] Petit Axe [___] [___],[___] [___] |
|  | Inguinal | ☐OUI ☐NON  ☐ Gauche ☐ Droite  ☐Visible et mesurable ☐Palpable et non mesurable  ☐Ferme ou ☐Souple  Rougeur ☐OUI ☐NON  Œdème des tissus ☐OUI ☐NON  Suppuration ☐OUI ☐NON  Score de douleur ____  Taille  (mm) : Grand Axe [___] [___],[___] [___] Petit Axe [___] [___],[___] [___] |
|  | Autre | ☐OUI ☐NON  ☐ Gauche ☐ Droite  ☐Visible et mesurable ☐Palpable et non mesurable  ☐Ferme ou ☐Souple  Rougeur ☐OUI ☐NON  Œdème des tissus ☐OUI ☐NON  Suppuration ☐OUI ☐NON  Score de douleur ____  Taille  (mm) : Grand Axe [___] [___],[___] [___] Petit Axe [___] [___],[___] [___] |
| **Vomissement** | ☐OUI ☐NON ☐Inconnu | |
| **Diarrhée** | ☐OUI ☐NON ☐Inconnu | |
| **Céphalées** | ☐OUI ☐NON ☐Inconnu | |
| **Crises convulsives** | ☐OUI ☐NON ☐Inconnu | |
| **Niveau de conscience**  **Echelle EODA (AVPU)** | ☐ Eveillé (conscience normale) ☐ Répond aux ordres ☐ Répond aux stimuli douloureux  ☐ Ne répond à aucun stimulus | |
| **PARAMETRES VITAUX** | | |
| **Mesures des signes vitaux – MATIN** ☐OUI ☐NON **Heure de l’évaluation :** [__] [__]/ [__] [__] | | |
| **Fréquence respiratoire** | [___] [___] cycles / min ☐NF | |
| **Fréquence cardiaque** | [___] [___] [___] pulsations / min ☐NF | |
| **Pression artérielle** | Systolique [___] [___] [___] / mmHg Diastolique [___] [___] [___]/ mmHg ☐NF | |
| **Température axillaire** | [___] [___]. [___] C ☐NF | |
| **Saturation d'oxygène** | [___] [___] % ☐en air ambiant ☐Sur oxygène supplémentaire [___] [___] L / min ☐NF  Débit d’oxygène [___] [___],[___] L / min ☐NF | |
| **Meures des signes vitaux – APRES MIDI** ☐OUI ☐NON **Heure de l’évaluation :** [__][__]/ [__] [__] | | |
| **Fréquence respiratoire** | [___] [___] cycles / min ☐NF | |
| **Fréquence cardiaque** | [___] [___] [___] pulsations / min ☐NF | |
| **Pression artérielle** | Systolique [___] [___] [___] / mmHg Diastolique [___] [___] [___]/ mmHg ☐NF | |
| **Température axillaire** | [___] [___]. [___] C ☐NF | |
| **Saturation d'oxygène** | [___] [___] % ☐en air ambiant ☐Sur oxygène supplémentaire [___] [___] L / min ☐NF  Débit d’oxygène [___] [___],[___] L / min ☐NF | |

1. **COLLECTE ECHANTILLLONS ET RESULTATS DES TESTS DE DIAGNOSTIC RAPIDES SUR SITE**

| **Prélèvement Bubon (Pus)** | ☐OUI ☐NON |
| --- | --- |
|  | Date de prélèvement [__][__]/ [__] [__] / [__] [__] [__] [__] |
|  | Résultat TDR peste (site) ☐Positif ☐Négatif |
|  | **N° Prélèvement BUBON** ECR \|__\| \|__\|__\| \|__\|__\| \|__\|__\|__\| \|__\|__\|__\| |
|  | **N° TLO :** |
| **Prélèvement crachat** | ☐OUI ☐NON |
|  | Date de prélèvement [__] [__]/ [__] [__] / [__] [__] [__] [__] |
|  | Résultat TDR peste (site) ☐Positif ☐Négatif |
|  | **N° Prélèvement CRACHAT** ECR \|__\| \|__\|__\| \|__\|__\| \|__\|__\|__\| \|__\|__\|__\| |
| **Prélèvement sanguin pour la sérologie** | ☐OUI ☐NON  Type de prélèvement pour la sérologie Sang capillaire☐ Sang veineux☐ |
|  | Date de prélèvement : [__][__]/ [__] [__] / [__] [__] [__] [__] |
|  | **N° Prélèvement SEROLOGIE** ECR \|__\| \|__\|__\| \|__\|__\| \|__\|__\|__\| \|__\|__\|__\| |
| **TDR paludisme** | ☐OUI ☐NON |
|  | Date du TDR paludisme [__][__]/ [__] [__] / [__] [__] [__] [__] |
|  | Résultat du TDR paludisme : ☐Positif ☐Négatif |

1. **PRISE QUOTIDIENNE MEDICAMENTEUSE – V1**

| **TRAITEMENT DE L’ETUDE** | | | | | |
| --- | --- | --- | --- | --- | --- |
| **Date de prise** [__] [__]/ [__] [__] / [__] [__] [__] [__] | | | | | |
| **Traitement dispensé a.m.** ☐OUI ☐NON, si OUI COMPLETER | | | | | |
| **Streptomycine IV a.m.** | | | ☐OUI  ☐NON ☐Inconnu | | Dose : [__][__][__][__] mg Heure de prise: [__][__]/ [__] [__]  Si la dose manquée, la raison : ________________________________  Si la dose modifiée, la raison : _________________________________  Si la dose refusée, la raison : __________________________________  Autre remarque sur la prise : |
| **Ciprofloxacine a.m.** | | | ☐OUI  ☐NON ☐Inconnu | | Dose: [__][__][__][__] mg Voie : ☐IV ☐PO Heure de prise : [__][__]/ [__] [__]  Si la dose manquée, la raison : __________________  Si la dose modifiée, la raison : __________________  Si la dose refusée, la raison : __________________  Si administration de ciprofloxacine IV, préciser la raison  ☐Impossibilité d’administrer la dose PO ☐Autre, précisez :  ______________________________________________________  Autre remarque sur la prise : |
| **Observation sur la prise : vomissements dans les 30 min après la prise** ☐OUI ☐NON, SI OUI COMPLETER | | | | | |
| **Ciprofloxacine ré-administrée (n°1)** ☐OUI ☐NON, Si NON préciser la raison...................................................Si OUI compléter : | | | | | |
|  |  | | | Dose: [__][__][__][__] mg Voie : ☐IV ☐PO Heure de prise : [__][__]/ [__] [__]  Si la dose manquée, la raison : __________________  Si la dose modifiée, la raison : __________________  Si la dose refusée, la raison : __________________  Si administration de ciprofloxacine IV, préciser la raison  ☐Impossibilité d’administrer la dose PO ☐Autre, précisez :  ______________________________________________________  Autre remarque sur la prise : | |
| **Observation sur la prise : vomissements dans les 30 min après la prise** ☐OUI ☐NON, SI OUI COMPLETER | | | | | |
| **Ciprofloxacine ré-administrée (n°2)** ☐OUI ☐NON, Si NON préciser la raison..................................................Si OUI compléter : | | | | | |
| **Ciprofloxacine a.m.** | | ☐OUI  ☐NON ☐Inconnu | | Dose: [__][__][__][__] mg Voie : ☐IV Heure de prise : [__][__]/ [__] [__]  Si la dose manquée, la raison : __________________  Si la dose modifiée, la raison : __________________  Si la dose refusée, la raison : __________________  Autre remarque sur la prise : | |
| **Traitement dispensé p.m.** ☐OUI ☐NON, si OUI COMPLETER | | | | | |
| **Streptomycine IV p.m.** | | | ☐OUI  ☐NON ☐Inconnu | | Dose : [__][__][__][__] mg Heure de prise : [__][__]/ [__] [__]  Si la dose manquée, la raison : ________________________________  Si la dose modifiée, la raison : _________________________________  Si la dose refusée, la raison : __________________________________  Autre remarque sur la prise : |
| **Ciprofloxacine p.m.** | | | ☐OUI  ☐NON ☐Inconnu | | Dose [__][__][__][__] mg Voie : ☐IV ☐PO Heure de prise: [__][__]/ [__] [__]  Si la dose manquée, la raison : __________________  Si la dose modifiée, la raison : __________________  Si la dose refusée, la raison : __________________  Autre remarque sur la prise : |
| **Observation sur la prise : vomissements dans les 30 min après la prise** ☐OUI ☐NON, SI OUI COMPLETER | | | | | |
| **Ciprofloxacine ré-administrée (n°1)** ☐OUI ☐NON, Si NON préciser la raison...................................................Si OUI compléter : | | | | | |
|  | | |  | | Dose: [__][__][__][__] mg Voie : ☐IV ☐PO Heure de prise : [__][__]/ [__] [__]  Si la dose manquée, la raison : __________________  Si la dose modifiée, la raison : __________________  Si la dose refusée, la raison : __________________  Si administration de ciprofloxacine IV, préciser la raison  ☐Impossibilité d’administrer la dose PO ☐Autre, précisez :  ______________________________________________________  Autre remarque sur la prise : |
| **Vomissements dans les 30 min après la prise** ☐OUI ☐NON, SI OUI COMPLETER | | | | | |
| **Ciprofloxacine ré-administrée (n°2)** ☐OUI ☐NON, Si NON préciser la raison...................................................Si OUI compléter : | | | | | |
| **Ciprofloxacine p.m.** | | | ☐OUI  ☐NON ☐Inconnu | | Dose: [__][__][__][__] mg Voie : ☐IV Heure de prise : [__][__]/ [__] [__]  Si la dose manquée, la raison : __________________  Si la dose modifiée, la raison : __________________  Si la dose refusée, la raison : __________________  Autre remarque sur la prise : |
| **TRAITEMENTS ALTERNATIFS ET AUTRES TRAITEMENTS** | | | | | |
| **Gentamicine a.m.** | | | ☐OUI  ☐NON ☐Inconnu | | Dose journalière : [__][__][__][__] mg Voie : ☐IV ☐ IM  Heure de prise: [__][__]/ [__] [__]  Si la dose manquée, la raison : __________________  Si la dose modifiée, la raison : __________________  Si la dose refusée, la raison : __________________ |
| **Gentamicine p.m.** | | | ☐OUI  ☐NON ☐Inconnu | | Dose journalière: [__][__][__][__] mg Voie : ☐IV ☐ IM  Heure de prise: [__][__]/ [__] [__]  Si la dose manquée, la raison : __________________  Si la dose modifiée, la raison : __________________  Si la dose refusée, la raison : __________________ |
| **Autres antibiotiques** | | | ☐OUI  ☐NON ☐Inconnu | | Nom 1: ________ _______________ ☐PO ☐IV ☐IM  Dose journalière [__][__][__][__] mg  Nom 2: ________________________ ☐PO ☐IV ☐IM  Dose journalière [__][__][__][__] mg  Nom 3: ______________________ ☐PO ☐IV ☐IM  Dose journalière [__][__][__][__] mg |
| **Antipaludiques** | | | ☐OUI  ☐NON ☐Inconnu | | Nom 4: ______________________ ☐PO ☐IV ☐IM  Dose journalière ______________  Nom 5: ____________ __________ ☐PO ☐IV ☐IM  Dose journalière ________________ |
| **Paracétamol** | | | ☐OUI ☐NON ☐Inconnu | | |
| **AINS** | | | ☐OUI ☐NON ☐Inconnu | | |
| **Corticostéroïdes** | | | ☐OUI ☐NON ☐Inconnu | | |
| **Fluides en IV** | | | ☐OUI ☐NON ☐Inconnu | | |

| **VISITE 2** |
| --- |

1. **Visite – V2**

| **Date prévisionnelle de la visite** | [__][__]/ [__] [__] / [__] [__] [__] [__] |
| --- | --- |
| **Jour de la visite** | 🞎J1 🞎J2 🞎J3 🞎J4  🞎J5 🞎J6 🞎J7 🞎J8 🞎J9 🞎J10  🞎J11 🞎J21  🞎M3 |
| **Lieu de la visite** | 🞎 Sur site 🞎 A domicile |
| **La visite a –t-elle pu être réalisée ?** | 🞎OUI 🞎NON |
| **Patient vu le jour de la visite, par :** | 🞎 Médecin / Responsable du site  🞎 TEC  🞎 Agent communautaire |
| **Date effective de la visite** | [__][__]/ [__] [__] / [__] [__] [__] [__] |
| **Déclaration évènement indésirable** | 🞎OUI, *si « oui » compléter le formulaire EI*  🞎NON |
| **Si la visite n’a pas pu être réalisée, précisez la raison :** | 🞎 Arrêt prématuré *(Compléter le formulaire « SORTIE prématurée/Fin d’étude »)*  🞎AUTRE, précisez la raison :**_____________________________________________________** |

1. **SYMPTOMES ET SIGNES CLINIQUES – V2**

| **EXAMEN CLINIQUE** | |
| --- | --- |
| **Date d’évaluation :** [__][__]/ [__] [__] / [__] [__] [__] [__] **Heure de l'évaluation :** [__][__]/ [__] [__] | |
| **Fièvre** | ☐OUI ☐NON ☐Inconnu |
| **Toux** | ☐OUI ☐NON ☐Inconnu |
| **Crachats purulents** | ☐OUI ☐NON ☐Inconnu |
| **Crachats sanglants** | ☐OUI ☐NON ☐Inconnu |
| **Hémoptysie franche** | ☐OUI ☐NON ☐Inconnu |
| **Dyspnée** | ☐OUI ☐NON ☐Inconnu |
| **Douleur thoracique** | ☐OUI ☐NON ☐Inconnu |
| **Vomissement** | ☐OUI ☐NON ☐Inconnu |
| **Diarrhée** | ☐OUI ☐NON ☐Inconnu |
| **Céphalées** | ☐OUI ☐NON ☐Inconnu |
| **Crises convulsives** | ☐OUI ☐NON ☐Inconnu |
| **Niveau de conscience**  **Echelle EODA (AVPU)** | ☐ Eveillé (conscience normale) ☐ Répond aux ordres ☐ Répond aux stimuli douloureux  ☐ Ne répond à aucun stimulus |
| **PARAMETRES VITAUX** | |
| **Mesures des signes vitaux – MATIN** ☐OUI ☐NON **Heure de l’évaluation :** [__] [__]/ [__] [__] | |
| **Fréquence respiratoire** | [___] [___] cycles / min ☐NF |
| **Fréquence cardiaque** | [___] [___] [___] pulsations / min ☐NF |
| **Pression artérielle** | Systolique [___] [___] [___] / mmHg Diastolique [___] [___] [___]/ mmHg ☐NF |
| **Température axillaire** | [___] [___]. [___] C ☐NF |
| **Saturation d'oxygène** | [___] [___] % ☐en air ambiant ☐Sur oxygène supplémentaire [___] [___] L / min ☐NF  Débit d’oxygène [___] [___],[___] L / min ☐NF |
| **Meures des signes vitaux – APRES MIDI** ☐OUI ☐NON **Heure de l’évaluation :** [__] [__]/ [__] [__] | |
| **Fréquence respiratoire** | [___] [___] cycles / min ☐NF |
| **Fréquence cardiaque** | [___] [___] [___] pulsations / min ☐NF |
| **Pression artérielle** | Systolique [___] [___] [___] / mmHg Diastolique [___] [___] [___]/ mmHg ☐NF |
| **Température axillaire** | [___] [___]. [___] C ☐NF |
| **Saturation d'oxygène** | [___] [___] % ☐en air ambiant ☐Sur oxygène supplémentaire [___] [___] L / min ☐NF  Débit d’oxygène [___] [___],[___] L / min ☐NF |

1. **PRISE QUOTIDIENNE DE MEDICAMENTS – V2**

| **TRAITEMENT DE L’ETUDE** | | | | | |
| --- | --- | --- | --- | --- | --- |
| **Date de prise** [__] [__]/ [__] [__] / [__] [__] [__] [__] | | | | | |
| **Traitement dispensé a.m.** ☐OUI ☐NON, si OUI COMPLETER | | | | | |
| **Streptomycine IV a.m.** | | | ☐OUI  ☐NON ☐Inconnu | | Dose : [__][__][__][__] mg Heure de prise: [__][__]/ [__] [__]  Si la dose manquée, la raison : ________________________________  Si la dose modifiée, la raison : _________________________________  Si la dose refusée, la raison : __________________________________  Autre remarque sur la prise : |
| **Ciprofloxacine a.m.** | | | ☐OUI  ☐NON ☐Inconnu | | Dose: [__][__][__][__] mg Voie : ☐IV ☐PO Heure de prise : [__][__]/ [__] [__]  Si la dose manquée, la raison : __________________  Si la dose modifiée, la raison : __________________  Si la dose refusée, la raison : __________________  Si administration de ciprofloxacine IV, préciser la raison  ☐Impossibilité d’administrer la dose PO ☐Autre, précisez :  ______________________________________________________  Autre remarque sur la prise : |
| **Observation sur la prise : vomissements dans les 30 min après la prise** ☐OUI ☐NON, SI OUI COMPLETER | | | | | |
| **Ciprofloxacine ré-administrée (n°1)** ☐OUI ☐NON, Si NON préciser la raison...................................................Si OUI compléter : | | | | | |
| **Ciprofloxacine a.m.** | ☐OUI  ☐NON ☐Inconnu | | | Dose: [__][__][__][__] mg Voie : ☐IV ☐PO Heure de prise : [__][__]/ [__] [__]  Si la dose manquée, la raison : __________________  Si la dose modifiée, la raison : __________________  Si la dose refusée, la raison : __________________  Si administration de ciprofloxacine IV, préciser la raison  ☐Impossibilité d’administrer la dose PO ☐Autre, précisez :  ______________________________________________________  Autre remarque sur la prise : | |
| **Observation sur la prise : vomissements dans les 30 min après la prise** ☐OUI ☐NON, SI OUI COMPLETER | | | | | |
| **Ciprofloxacine ré-administrée (n°2)** ☐OUI ☐NON, Si NON préciser la raison..................................................Si OUI compléter : | | | | | |
| **Ciprofloxacine a.m.** | | ☐OUI  ☐NON ☐Inconnu | | Dose: [__][__][__][__] mg Voie : ☐IV Heure de prise : [__][__]/ [__] [__]  Si la dose manquée, la raison : __________________  Si la dose modifiée, la raison : __________________  Si la dose refusée, la raison : __________________  Autre remarque sur la prise : | |
| **Traitement dispensé p.m.** ☐OUI ☐NON, si OUI COMPLETER | | | | | |
| **Streptomycine IV p.m.** | | | ☐OUI  ☐NON ☐Inconnu | | Dose : [__][__][__][__] mg Heure de prise : [__][__]/ [__] [__]  Si la dose manquée, la raison : ________________________________  Si la dose modifiée, la raison : _________________________________  Si la dose refusée, la raison : __________________________________  Autre remarque sur la prise : |
| **Ciprofloxacine p.m.** | | | ☐OUI  ☐NON ☐Inconnu | | Dose [__][__][__][__] mg Voie : ☐IV ☐PO Heure de prise: [__][__]/ [__] [__]  Si la dose manquée, la raison : __________________  Si la dose modifiée, la raison : __________________  Si la dose refusée, la raison : __________________  Si administration de ciprofloxacine IV, préciser la raison  ☐Impossibilité d’administrer la dose PO ☐Autre, précisez :  ______________________________________________________  Autre remarque sur la prise : |
| **Observation sur la prise : vomissements dans les 30 min après la prise** ☐OUI ☐NON, SI OUI COMPLETER | | | | | |
| **Ciprofloxacine ré-administrée (n°1)** ☐OUI ☐NON, Si NON préciser la raison...................................................Si OUI compléter : | | | | | |
| **Ciprofloxacine p.m.** | | | ☐OUI  ☐NON ☐Inconnu | | Dose: [__][__][__][__] mg Voie : ☐IV ☐PO Heure de prise : [__][__]/ [__] [__]  Si la dose manquée, la raison : __________________  Si la dose modifiée, la raison : __________________  Si la dose refusée, la raison : __________________  Si administration de ciprofloxacine IV, préciser la raison  ☐Impossibilité d’administrer la dose PO ☐Autre, précisez :  ______________________________________________________  Autre remarque sur la prise : |
| **Vomissements dans les 30 min après la prise** ☐OUI ☐NON, SI OUI COMPLETER | | | | | |
| **Ciprofloxacine ré-administrée (n°2)** ☐OUI ☐NON, Si NON préciser la raison...................................................Si OUI compléter : | | | | | |
| **Ciprofloxacine p.m.** | | | ☐OUI  ☐NON ☐Inconnu | | Dose: [__][__][__][__] mg Voie : ☐IV Heure de prise : [__][__]/ [__] [__]  Si la dose manquée, la raison : __________________  Si la dose modifiée, la raison : __________________  Si la dose refusée, la raison : __________________  Autre remarque sur la prise : |
| **TRAITEMENTS ALTERNATIFS ET AUTRES TRAITEMENTS** | | | | | |
| **Gentamicine a.m.** | | | ☐OUI  ☐NON ☐Inconnu | | Dose journalière : [__][__][__][__] mg Voie : ☐IV ☐ IM  Heure de prise: [__][__]/ [__] [__]  Si la dose manquée, la raison : __________________  Si la dose modifiée, la raison : __________________  Si la dose refusée, la raison : __________________ |
| **Gentamicine p.m.** | | | ☐OUI  ☐NON ☐Inconnu | | Dose journalière: [__][__][__][__] mg Voie : ☐IV ☐ IM  Heure de prise: [__][__]/ [__] [__]  Si la dose manquée, la raison : __________________  Si la dose modifiée, la raison : __________________  Si la dose refusée, la raison : __________________ |
| **Autres antibiotiques** | | | ☐OUI  ☐NON ☐Inconnu | | Nom 1: ________ _______________ ☐PO ☐IV ☐IM  Dose journalière [__][__][__][__] mg  Nom 2: ________________________ ☐PO ☐IV ☐IM  Dose journalière [__][__][__][__] mg  Nom 3: ______________________ ☐PO ☐IV ☐IM  Dose journalière [__][__][__][__] mg |
| **Antipaludiques** | | | ☐OUI  ☐NON ☐Inconnu | | Nom 4: ______________________ ☐PO ☐IV ☐IM  Dose journalière ______________  Nom 5: ____________ __________ ☐PO ☐IV ☐IM  Dose journalière ________________ |
| **Paracétamol** | | | ☐OUI ☐NON ☐Inconnu | | |
| **AINS** | | | ☐OUI ☐NON ☐Inconnu | | |
| **Corticostéroïdes** | | | ☐OUI ☐NON ☐Inconnu | | |
| **Fluides en IV** | | | ☐OUI ☐NON ☐Inconnu | | |

| **VISITE 3** |
| --- |

1. **Visite – V3**

| **Date prévisionnelle de la visite** | [__][__]/ [__] [__] / [__] [__] [__] [__] |
| --- | --- |
| **Jour de la visite** | 🞎J1 🞎J2 🞎J3 🞎J4  🞎J5 🞎J6 🞎J7 🞎J8 🞎J9 🞎J10  🞎J11 🞎J21  🞎M3 |
| **Lieu de la visite** | 🞎 Sur site 🞎 A domicile |
| **La visite a –t-elle pu être réalisée ?** | 🞎OUI 🞎NON |
| **Patient vu le jour de la visite, par :** | 🞎 Médecin / Responsable du site  🞎 TEC  🞎 Agent communautaire |
| **Date effective de la visite** | [__][__]/ [__] [__] / [__] [__] [__] [__] |
| **Déclaration évènement indésirable** | 🞎OUI, *si « oui » compléter le formulaire EI*  🞎NON |
| **Si la visite n’a pas pu être réalisée, précisez la raison :** | 🞎 Arrêt prématuré  *(Compléter le formulaire « SORTIE prématurée/Fin d’étude »)*  🞎AUTRE, précisez la raison :**_____________________________________________________** |

1. **SYMPTOMES ET SIGNES CLINIQUES – V3**

| **EXAMEN CLINIQUE** | |
| --- | --- |
| **Date d’évaluation :** [__] [__]/ [__] [__] / [__] [__] [__] [__] **Heure de l'évaluation :** [__][__]/ [__] [__] | |
| **Fièvre** | ☐OUI ☐NON ☐Inconnu |
| **Toux** | ☐OUI ☐NON ☐Inconnu |
| **Crachats purulents** | ☐OUI ☐NON ☐Inconnu |
| **Crachats sanglants** | ☐OUI ☐NON ☐Inconnu |
| **Hémoptysie franche** | ☐OUI ☐NON ☐Inconnu |
| **Dyspnée** | ☐OUI ☐NON ☐Inconnu |
| **Douleur thoracique** | ☐OUI ☐NON ☐Inconnu |
| **Vomissement** | ☐OUI ☐NON ☐Inconnu |
| **Diarrhée** | ☐OUI ☐NON ☐Inconnu |
| **Céphalées** | ☐OUI ☐NON ☐Inconnu |
| **Crises convulsives** | ☐OUI ☐NON ☐Inconnu |
| **Niveau de conscience**  **Echelle EODA (AVPU)** | ☐ Eveillé (conscience normale) ☐ Répond aux ordres ☐ Répond aux stimuli douloureux  ☐ Ne répond à aucun stimulus |
| **PARAMETRES VTAUX** | |
| **Mesures des signes vitaux – MATIN** ☐OUI ☐NON **Heure de l’évaluation :** [__][__]/ [__] [__] | |
| **Fréquence respiratoire** | [___] [___] cycles / min ☐NF |
| **Fréquence cardiaque** | [___] [___] [___] pulsations / min ☐NF |
| **Pression artérielle** | Systolique [___] [___] [___] / mmHg Diastolique [___] [___] [___]/ mmHg ☐NF |
| **Température axillaire** | [___] [___]. [___] C ☐NF |
| **Saturation d'oxygène** | [___] [___] % ☐en air ambiant ☐Sur oxygène supplémentaire [___] [___] L / min ☐NF  Débit d’oxygène [___] [___],[___] L / min ☐NF |
| **Meures des signes vitaux – APRES MIDI** ☐OUI ☐NON **Heure de l’évaluation :** [__][__]/ [__] [__] | |
| **Fréquence respiratoire** | [___] [___] cycles / min ☐NF |
| **Fréquence cardiaque** | [___] [___] [___] pulsations / min ☐NF |
| **Pression artérielle** | Systolique [___] [___] [___] / mmHg Diastolique [___] [___] [___]/ mmHg ☐NF |
| **Température axillaire** | [___] [___]. [___] C ☐NF |
| **Saturation d'oxygène** | [___] [___] % ☐en air ambiant ☐Sur oxygène supplémentaire [___] [___] L / min ☐NF  Débit d’oxygène [___] [___],[___] L / min ☐NF |

1. **PRISE QUOTIDIENNE DE MEDICAMENTS – V3**

| **TRAITEMENT DE L’ETUDE** | | | | | |
| --- | --- | --- | --- | --- | --- |
| **Date de prise** [__] [__]/ [__] [__] / [__] [__] [__] [__] | | | | | |
| **Traitement dispensé a.m.** ☐OUI ☐NON, si OUI COMPLETER | | | | | |
| **Streptomycine IV a.m.** | | | ☐OUI  ☐NON ☐Inconnu | | Dose : [__][__][__][__] mg Heure de prise: [__][__]/ [__] [__]  Si la dose manquée, la raison : ________________________________  Si la dose modifiée, la raison : _________________________________  Si la dose refusée, la raison : __________________________________  Autre remarque sur la prise : |
| **Ciprofloxacine a.m.** | | | ☐OUI  ☐NON ☐Inconnu | | Dose: [__][__][__][__] mg Voie : ☐IV ☐PO Heure de prise : [__][__]/ [__] [__]  Si la dose manquée, la raison : __________________  Si la dose modifiée, la raison : __________________  Si la dose refusée, la raison : __________________  Si administration de ciprofloxacine IV, préciser la raison  ☐Impossibilité d’administrer la dose PO ☐Autre, précisez :  ______________________________________________________  Autre remarque sur la prise : |
| **Observation sur la prise : vomissements dans les 30 min après la prise** ☐OUI ☐NON, SI OUI COMPLETER | | | | | |
| **Ciprofloxacine ré-administrée (n°1)** ☐OUI ☐NON, Si NON préciser la raison...................................................Si OUI compléter : | | | | | |
|  |  | | | Dose: [__][__][__][__] mg Voie : ☐IV ☐PO Heure de prise : [__][__]/ [__] [__]  Si la dose manquée, la raison : __________________  Si la dose modifiée, la raison : __________________  Si la dose refusée, la raison : __________________  Si administration de ciprofloxacine IV, préciser la raison  ☐Impossibilité d’administrer la dose PO ☐Autre, précisez :  ______________________________________________________  Autre remarque sur la prise : | |
| **Observation sur la prise : vomissements dans les 30 min après la prise** ☐OUI ☐NON, SI OUI COMPLETER | | | | | |
| **Ciprofloxacine ré-administrée (n°2)** ☐OUI ☐NON, Si NON préciser la raison..................................................Si OUI compléter : | | | | | |
|  | |  | | Dose: [__][__][__][__] mg Voie : ☐IV Heure de prise : [__][__]/ [__] [__]  Si la dose manquée, la raison : __________________  Si la dose modifiée, la raison : __________________  Si la dose refusée, la raison : __________________  Autre remarque sur la prise : | |
| **Traitement dispensé p.m.** ☐OUI ☐NON, si OUI COMPLETER | | | | | |
| **Streptomycine IV p.m.** | | | ☐OUI  ☐NON ☐Inconnu | | Dose : [__][__][__][__] mg Heure de prise : [__][__]/ [__] [__]  Si la dose manquée, la raison : ________________________________  Si la dose modifiée, la raison : _________________________________  Si la dose refusée, la raison : __________________________________  Autre remarque sur la prise : |
| **Ciprofloxacine p.m.** | | | ☐OUI  ☐NON ☐Inconnu | | Dose [__][__][__][__] mg Voie : ☐IV ☐PO Heure de prise: [__][__]/ [__] [__]  Si la dose manquée, la raison : __________________  Si la dose modifiée, la raison : __________________  Si la dose refusée, la raison : __________________  Si administration de ciprofloxacine IV, préciser la raison  ☐Impossibilité d’administrer la dose PO ☐Autre, précisez :  ______________________________________________________  Autre remarque sur la prise : |
| **Observation sur la prise : vomissements dans les 30 min après la prise** ☐OUI ☐NON, SI OUI COMPLETER | | | | | |
| **Ciprofloxacine ré-administrée (n°1)** ☐OUI ☐NON, Si NON préciser la raison...................................................Si OUI compléter : | | | | | |
| **Ciprofloxacine p.m.** | | | ☐OUI  ☐NON ☐Inconnu | | Dose: [__][__][__][__] mg Voie : ☐IV ☐PO Heure de prise : [__][__]/ [__] [__]  Si la dose manquée, la raison : __________________  Si la dose modifiée, la raison : __________________  Si la dose refusée, la raison : __________________ Si administration de ciprofloxacine IV, préciser la raison  ☐Impossibilité d’administrer la dose PO ☐Autre, précisez :  ______________________________________________________  Autre remarque sur la prise : |
| **Vomissements dans les 30 min après la prise** ☐OUI ☐NON, SI OUI COMPLETER | | | | | |
| **Ciprofloxacine ré-administrée (n°2)** ☐OUI ☐NON, Si NON préciser la raison...................................................Si OUI compléter : | | | | | |
| **Ciprofloxacine p.m.** | | | ☐OUI  ☐NON ☐Inconnu | | Dose: [__][__][__][__] mg Voie : ☐IV Heure de prise : [__][__]/ [__] [__]  Si la dose manquée, la raison : __________________  Si la dose modifiée, la raison : __________________  Si la dose refusée, la raison : __________________  Autre remarque sur la prise : |
| **TRAITEMENTS ALTERNATIFS ET AUTRES TRAITEMENTS** | | | | | |
| **Gentamicine a.m.** | | | ☐OUI  ☐NON ☐Inconnu | | Dose journalière : [__][__][__][__] mg Voie : ☐IV ☐ IM  Heure de prise: [__][__]/ [__] [__]  Si la dose manquée, la raison : __________________  Si la dose modifiée, la raison : __________________  Si la dose refusée, la raison : __________________ |
| **Gentamicine p.m.** | | | ☐OUI  ☐NON ☐Inconnu | | Dose journalière: [__][__][__][__] mg Voie : ☐IV ☐ IM  Heure de prise: [__][__]/ [__] [__]  Si la dose manquée, la raison : __________________  Si la dose modifiée, la raison : __________________  Si la dose refusée, la raison : __________________ |
| **Autres antibiotiques** | | | ☐OUI  ☐NON ☐Inconnu | | Nom 1: ________ _______________ ☐PO ☐IV ☐IM  Dose journalière [__][__][__][__] mg  Nom 2: ________________________ ☐PO ☐IV ☐IM  Dose journalière [__][__][__][__] mg  Nom 3: ______________________ ☐PO ☐IV ☐IM  Dose journalière [__][__][__][__] mg |
| **Antipaludiques** | | | ☐OUI  ☐NON ☐Inconnu | | Nom 4: ______________________ ☐PO ☐IV ☐IM  Dose journalière ______________  Nom 5: ____________ __________ ☐PO ☐IV ☐IM  Dose journalière ________________ |
| **Paracétamol** | | | ☐OUI ☐NON ☐Inconnu | | |
| **AINS** | | | ☐OUI ☐NON ☐Inconnu | | |
| **Corticostéroïdes** | | | ☐OUI ☐NON ☐Inconnu | | |
| **Fluides en IV** | | | ☐OUI ☐NON ☐Inconnu | | |

| **VISITE 4** |
| --- |

1. **Visite - V4**

| **Date prévisionnelle de la visite** | [__][__]/ [__] [__] / [__] [__] [__] [__] |
| --- | --- |
| **Jour de la visite** | 🞎J1 🞎J2 🞎J3 🞎J4  🞎J5 🞎J6 🞎J7 🞎J8 🞎J9 🞎J10  🞎J11 🞎J21  🞎M3 |
| **Lieu de la visite** | 🞎 Sur site 🞎 A domicile |
| **La visite a –t-elle pu être réalisée ?** | 🞎OUI 🞎NON |
| **Patient vu le jour de la visite, par :** | 🞎 Médecin / Responsable du site  🞎 TEC  🞎 Agent communautaire |
| **Date effective de la visite** | [__][__]/ [__] [__] / [__] [__] [__] [__] |
| **Déclaration évènement indésirable** | 🞎OUI, *si « oui » compléter le formulaire EI*  🞎NON |
| **Si la visite n’a pas pu être réalisée, précisez la raison :** | 🞎 Arrêt prématuré  *(Compléter le formulaire « SORTIE prématurée/Fin d’étude »)*  🞎AUTRE, précisez la raison :**_____________________________________________________** |

1. **SYMPTOMES ET SIGNES CLINIQUES – V4**

| **EXAMEN CLINIQUE** | | |
| --- | --- | --- |
| **Date d’évaluation :** [__][__]/ [__] [__] / [__] [__] [__] [__] **Heure de l'évaluation :** [__][__]/ [__] [__] | | |
| **Fièvre** | ☐OUI ☐NON ☐Inconnu | |
| **Toux** | ☐OUI ☐NON ☐Inconnu | |
| **Crachats purulents** | ☐OUI ☐NON ☐Inconnu | |
| **Crachats sanglants** | ☐OUI ☐NON ☐Inconnu | |
| **Hémoptysie franche** | ☐OUI ☐NON ☐Inconnu | |
| **Dyspnée** | ☐OUI ☐NON ☐Inconnu | |
| **Douleur thoracique** | ☐OUI ☐NON ☐Inconnu | |
| **Bubon(s)** | ☐OUI Depuis combien de temps en jours [__] [__] Nombre de bubons  [__][__]  ☐NON ☐Inconnu | |
| **Site du Bubon(s)** | ☐Axillaire | ☐OUI ☐NON  ☐ Gauche ☐ Droite  ☐Visible et mesurable ☐Palpable et non mesurable  ☐Ferme ou ☐Souple  Rougeur ☐OUI ☐NON  Œdème des tissus ☐OUI ☐NON  Suppuration ☐OUI ☐NON  Score de douleur ____  Taille  (mm) : Grand Axe [___] [___],[___] [___] Petit Axe [___] [___],[___] [___] |
|  | ☐Cervical | ☐OUI ☐NON  ☐ Gauche ☐ Droite  ☐Visible et mesurable ☐Palpable et non mesurable  ☐Ferme ou ☐Souple  Rougeur ☐OUI ☐NON  Œdème des tissus ☐OUI ☐NON  Suppuration ☐OUI ☐NON  Score de douleur ____  Taille  (mm) : Grand Axe [___] [___],[___] [___] Petit Axe [___] [___],[___] [___] |
|  | ☐Inguinal | ☐OUI ☐NON  ☐ Gauche ☐ Droite  ☐Visible et mesurable ☐Palpable et non mesurable  ☐Ferme ou ☐Souple  Rougeur ☐OUI ☐NON  Œdème des tissus ☐OUI ☐NON  Suppuration ☐OUI ☐NON  Score de douleur ____  Taille  (mm) : Grand Axe [___] [___],[___] [___] Petit Axe [___] [___],[___] [___] |
|  | ☐Autre | ☐OUI ☐NON  ☐ Gauche ☐ Droite  ☐Visible et mesurable ☐Palpable et non mesurable  ☐Ferme ou ☐Souple  Rougeur ☐OUI ☐NON  Œdème des tissus ☐OUI ☐NON  Suppuration ☐OUI ☐NON  Score de douleur ____  Taille  (mm) : Grand Axe [___] [___],[___] [___] Petit Axe [___] [___],[___] [___] |
| **Vomissement** | ☐OUI ☐NON ☐Inconnu | |
| **Diarrhée** | ☐OUI ☐NON ☐Inconnu | |
| **Céphalées** | ☐OUI ☐NON ☐Inconnu | |
| **Crises convulsives** | ☐OUI ☐NON ☐Inconnu | |
| **Niveau de conscience**  **Echelle EODA (AVPU)** | ☐ Eveillé (conscience normale) ☐ Répond aux ordres ☐ Répond aux stimuli douloureux  ☐ Ne répond à aucun stimulus | |
| **PARAMETRES VITAUX** | | |
| **Mesures des signes vitaux – MATIN** ☐OUI ☐NON **Heure de l’évaluation :** [__] [__]/ [__] [__] | | |
| **Fréquence respiratoire** | [___] [___] cycles / min ☐NF | |
| **Fréquence cardiaque** | [___] [___] [___] pulsations / min ☐NF | |
| **Pression artérielle** | Systolique [___] [___] [___] / mmHg Diastolique [___] [___] [___]/ mmHg ☐NF | |
| **Température axillaire** | [___] [___]. [___] C ☐NF | |
| **Saturation d'oxygène** | [___] [___] % ☐en air ambiant ☐Sur oxygène supplémentaire [___] [___] L / min ☐NF  Débit d’oxygène [___] [___],[___] L / min ☐NF | |
| **Meures des signes vitaux – APRES MIDI** ☐OUI ☐NON **Heure de l’évaluation :** [__][__]/ [__] [__] | | |
| **Fréquence respiratoire** | [___] [___] cycles / min ☐NF | |
| **Fréquence cardiaque** | [___] [___] [___] pulsations / min ☐NF | |
| **Pression artérielle** | Systolique [___] [___] [___] / mmHg Diastolique [___] [___] [___]/ mmHg ☐NF | |
| **Température axillaire** | [___] [___]. [___] C ☐NF | |
| **Saturation d'oxygène** | [___] [___] % ☐en air ambiant ☐Sur oxygène supplémentaire [___] [___] L / min ☐NF  Débit d’oxygène [___] [___],[___] L / min ☐NF | |

1. **PRISE QUOTIDIENNE DE MEDICAMENTS – V4**

| **TRAITEMENT DE L’ETUDE** | | | | | |
| --- | --- | --- | --- | --- | --- |
| **Date de prise** [__] [__]/ [__] [__] / [__] [__] [__] [__] | | | | | |
| **Traitement dispensé a.m.** ☐OUI ☐NON, si OUI COMPLETER | | | | | |
| **Streptomycine IV a.m.** | | | ☐OUI  ☐NON ☐Inconnu | | Dose : [__][__][__][__] mg Heure de prise: [__][__]/ [__] [__]  Si la dose manquée, la raison : ________________________________  Si la dose modifiée, la raison : _________________________________  Si la dose refusée, la raison : __________________________________  Autre remarque sur la prise : |
| **Ciprofloxacine a.m.** | | | ☐OUI  ☐NON ☐Inconnu | | Dose: [__][__][__][__] mg Voie : ☐IV ☐PO Heure de prise : [__][__]/ [__] [__]  Si la dose manquée, la raison : __________________  Si la dose modifiée, la raison : __________________  Si la dose refusée, la raison : __________________  Si administration de ciprofloxacine IV, préciser la raison  ☐Impossibilité d’administrer la dose PO ☐Autre, précisez :  ______________________________________________________  Autre remarque sur la prise : |
| **Observation sur la prise : vomissements dans les 30 min après la prise** ☐OUI ☐NON, SI OUI COMPLETER | | | | | |
| **Ciprofloxacine ré-administrée (n°1)** ☐OUI ☐NON, Si NON préciser la raison...................................................Si OUI compléter : | | | | | |
| **Ciprofloxacine a.m.** | ☐OUI  ☐NON ☐Inconnu | | | Dose: [__][__][__][__] mg Voie : ☐IV ☐PO Heure de prise : [__][__]/ [__] [__]  Si la dose manquée, la raison : __________________  Si la dose modifiée, la raison : __________________  Si la dose refusée, la raison : __________________  Si administration de ciprofloxacine IV, préciser la raison  ☐Impossibilité d’administrer la dose PO ☐Autre, précisez :  ______________________________________________________  Autre remarque sur la prise : | |
| **Observation sur la prise : vomissements dans les 30 min après la prise** ☐OUI ☐NON, SI OUI COMPLETER | | | | | |
| **Ciprofloxacine ré-administrée (n°2)** ☐OUI ☐NON, Si NON préciser la raison..................................................Si OUI compléter : | | | | | |
| **Ciprofloxacine a.m.** | | ☐OUI  ☐NON ☐Inconnu | | Dose: [__][__][__][__] mg Voie : ☐IV Heure de prise : [__][__]/ [__] [__]  Si la dose manquée, la raison : __________________  Si la dose modifiée, la raison : __________________  Si la dose refusée, la raison : __________________  Autre remarque sur la prise : | |
| **Traitement dispensé p.m.** ☐OUI ☐NON, si OUI COMPLETER | | | | | |
| **Streptomycine IV p.m.** | | | ☐OUI  ☐NON ☐Inconnu | | Dose : [__][__][__][__] mg Heure de prise : [__][__]/ [__] [__]  Si la dose manquée, la raison : ________________________________  Si la dose modifiée, la raison : _________________________________  Si la dose refusée, la raison : __________________________________  Autre remarque sur la prise : |
| **Ciprofloxacine p.m.** | | | ☐OUI  ☐NON ☐Inconnu | | Dose [__][__][__][__] mg Voie : ☐IV ☐PO Heure de prise: [__][__]/ [__] [__]  Si la dose manquée, la raison : __________________  Si la dose modifiée, la raison : __________________  Si la dose refusée, la raison : __________________  Autre remarque sur la prise : |
| **Observation sur la prise : vomissements dans les 30 min après la prise** ☐OUI ☐NON, SI OUI COMPLETER | | | | | |
| **Ciprofloxacine ré-administrée (n°1)** ☐OUI ☐NON, Si NON préciser la raison...................................................Si OUI compléter : | | | | | |
| **Ciprofloxacine p.m.** | | | ☐OUI  ☐NON ☐Inconnu | | Dose: [__][__][__][__] mg Voie : ☐IV ☐PO Heure de prise : [__][__]/ [__] [__]  Si la dose manquée, la raison : __________________  Si la dose modifiée, la raison : __________________  Si la dose refusée, la raison : __________________  Si administration de ciprofloxacine IV, préciser la raison  ☐Impossibilité d’administrer la dose PO ☐Autre, précisez :  ______________________________________________________  Autre remarque sur la prise : |
| **Vomissements dans les 30 min après la prise** ☐OUI ☐NON, SI OUI COMPLETER | | | | | |
| **Ciprofloxacine ré-administrée (n°2)** ☐OUI ☐NON, Si NON préciser la raison...................................................Si OUI compléter : | | | | | |
| **Ciprofloxacine p.m.** | | | ☐OUI  ☐NON ☐Inconnu | | Dose: [__][__][__][__] mg Voie : ☐IV Heure de prise : [__][__]/ [__] [__]  Si la dose manquée, la raison : __________________  Si la dose modifiée, la raison : __________________  Si la dose refusée, la raison : __________________  Autre remarque sur la prise : |
| **TRAITEMENTS ALTERNATIFS ET AUTRES TRAITEMENTS** | | | | | |
| **Gentamicine a.m.** | | | ☐OUI  ☐NON ☐Inconnu | | Dose journalière : [__][__][__][__] mg Voie : ☐IV ☐ IM  Heure de prise: [__][__]/ [__] [__]  Si la dose manquée, la raison : __________________  Si la dose modifiée, la raison : __________________  Si la dose refusée, la raison : __________________ |
| **Gentamicine p.m.** | | | ☐OUI  ☐NON ☐Inconnu | | Dose journalière: [__][__][__][__] mg Voie : ☐IV ☐ IM  Heure de prise: [__][__]/ [__] [__]  Si la dose manquée, la raison : __________________  Si la dose modifiée, la raison : __________________  Si la dose refusée, la raison : __________________ |
| **Autres antibiotiques** | | | ☐OUI  ☐NON ☐Inconnu | | Nom 1: ________ _______________ ☐PO ☐IV ☐IM  Dose journalière [__][__][__][__] mg  Nom 2: ________________________ ☐PO ☐IV ☐IM  Dose journalière [__][__][__][__] mg  Nom 3: ______________________ ☐PO ☐IV ☐IM  Dose journalière [__][__][__][__] mg |
| **Antipaludiques** | | | ☐OUI  ☐NON ☐Inconnu | | Nom 4: ______________________ ☐PO ☐IV ☐IM  Dose journalière ______________  Nom 5: ____________ __________ ☐PO ☐IV ☐IM  Dose journalière ________________ |
| **Paracétamol** | | | ☐OUI ☐NON ☐Inconnu | | |
| **AINS** | | | ☐OUI ☐NON ☐Inconnu | | |
| **Corticostéroïdes** | | | ☐OUI ☐NON ☐Inconnu | | |
| **Fluides en IV** | | | ☐OUI ☐NON ☐Inconnu | | |

| **VISITE 5** |
| --- |

1. **VISITE - V5**

| **Date prévisionnelle de la visite** | [__][__]/ [__] [__] / [__] [__] [__] [__] |
| --- | --- |
| **Jour de la visite** | 🞎J1 🞎J2 🞎J3 🞎J4  🞎J5 🞎J6 🞎J7 🞎J8 🞎J9 🞎J10  🞎J11 🞎J21  🞎M3 |
| **Lieu de la visite** | 🞎 Sur site 🞎 A domicile |
| **La visite a –t-elle pu être réalisée ?** | 🞎OUI 🞎NON |
| **Patient vu le jour de la visite, par :** | 🞎 Médecin / Responsable du site  🞎 TEC  🞎 Agent communautaire |
| **Date effective de la visite** | [__] [__] / [__] [__] / [__] [__] [__] [__] |
| **Déclaration évènement indésirable** | 🞎OUI, *si « oui » compléter le formulaire EI*  🞎NON |
| **Si la visite n’a pas pu être réalisée, précisez la raison :** | 🞎 Arrêt prématuré  *(Compléter le formulaire « SORTIE prématurée/Fin d’étude »)*  🞎AUTRE, précisez la raison :**_____________________________________________________** |

1. **PRISE QUOTIDIENNE DE MEDICAMENTS – V5** *(données transcrites à partir de la fiche d’observance complétée par les agents communautaires)*

| **TRAITEMENT DE L’ETUDE** | | |
| --- | --- | --- |
| **Date de la visite** [__][__]/ [__] [__] / [__] [__] [__] [__] | | |
| **Traitement dispensé** ☐OUI ☐NON, si OUI COMPLETER | | |
| **Ciprofloxacine a.m.** | ☐OUI  ☐NON  ☐Inconnu | Dose: [__][__][__][__] mg Voie : ☐IV ☐PO  Dose non prise / vomissements / aggravation de l’état ☐OUI ☐NON  Si OUI Référer le patient au CSB  Patient référé au CSB ☐OUI ☐NON |
| **Ciprofloxacine p.m.** | ☐OUI  ☐NON  ☐Inconnu | Dose [__][__][__][__] mg Voie : ☐IV ☐PO  Dose non prise / vomissements / aggravation de l’état ☐OUI ☐NON  Si OUI Référer le patient au CSB  Patient référé au CSB ☐OUI ☐NON |
| **Streptomycine IV a.m** | ☐OUI  ☐NON  ☐Inconnu | Dose: [__][__][__][__] mg |
| **Streptomycine IV p.m** | ☐OUI  ☐NON  ☐Inconnu | Dose: [__][__][__][__] mg |

| **VISITE 6** |
| --- |

1. **VISITE – V6**

| **Date prévisionnelle de la visite** | [__][__]/ [__] [__] / [__] [__] [__] [__] |
| --- | --- |
| **Jour de la visite** | 🞎J1 🞎J2 🞎J3 🞎J4  🞎J5 🞎J6 🞎J7 🞎J8 🞎J9 🞎J10  🞎J11 🞎J21  🞎M3 |
| **Lieu de la visite** | 🞎 Sur site 🞎 A domicile |
| **La visite a –t-elle pu être réalisée ?** | 🞎OUI 🞎NON |
| **Patient vu le jour de la visite, par :** | 🞎 Médecin / Responsable du site  🞎 TEC  🞎 Agent communautaire |
| **Date effective de la visite** | [__][__]/ [__] [__] / [__] [__] [__] [__] |
| **Déclaration évènement indésirable** | 🞎OUI, *si « oui » compléter le formulaire EI*  🞎NON |
| **Si la visite n’a pas pu être réalisée, précisez la raison :** | 🞎 Arrêt prématuré  *(Compléter le formulaire « SORTIE prématurée/Fin d’étude »)*  🞎AUTRE, précisez la raison :**_____________________________________________________** |

1. **PRISE QUOTIDIENNE DE MEDICAMENTS – V6** (données transcrites à partir de la fiche d’observance complétée par les agents communautaires)

| **TRAITEMENT DE L’ETUDE** | | |
| --- | --- | --- |
| **Date de la visite** [__][__]/ [__] [__] / [__] [__] [__] [__] | | |
| **Traitement dispensé** ☐OUI ☐NON, si OUI COMPLETER | | |
| **Ciprofloxacine a.m.** | ☐OUI  ☐NON  ☐Inconnu | Dose: [__][__][__][__] mg Voie : ☐IV ☐PO  Si patient suivi à domicile par l’AC :  Dose non prise / vomissements / aggravation de l’état ☐OUI ☐NON  Si OUI Référer le patient au CSB : Patient référé au CSB ☐OUI ☐NON  Si patient suivi au sein de la structure sanitaire :  Remarque sur la prise : |
| **Streptomycine IV a.m** | ☐OUI  ☐NON  ☐Inconnu | Dose [__][__][__][__] mg |
| **Ciprofloxacine p.m.** | ☐OUI  ☐NON  ☐Inconnu | Dose [__][__][__][__] mg Voie : ☐IV ☐PO  Si patient suivi à domicile par l’AC :  Dose non prise / vomissements / aggravation de l’état ☐OUI ☐NON  Si OUI Référer le patient au CSB : Patient référé au CSB ☐OUI ☐NON  Si patient suivi au sein de la structure sanitaire :  Remarque sur la prise : |

| **VISITE 7** |
| --- |

1. **VISITE – V7**

| **Date prévisionnelle de la visite** | [__][__]/ [__] [__] / [__] [__] [__] [__] |
| --- | --- |
| **Jour de la visite** | 🞎J1 🞎J2 🞎J3 🞎J4  🞎J5 🞎J6 🞎J7 🞎J8 🞎J9 🞎J10  🞎J11 🞎J21  🞎M3 |
| **Lieu de la visite** | 🞎 Sur site 🞎 A domicile |
| **La visite a –t-elle pu être réalisée ?** | 🞎OUI 🞎NON |
| **Patient vu le jour de la visite, par :** | 🞎 Médecin / Responsable du site  🞎 TEC  🞎 Agent communautaire |
| **Date effective de la visite** | [__][__]/ [__] [__] / [__] [__] [__] [__] |
| **Déclaration évènement indésirable** | 🞎OUI, *si « oui » compléter le formulaire EI*  🞎NON |
| **Si la visite n’a pas pu être réalisée, précisez la raison :** | 🞎 Arrêt prématuré  *(Compléter le formulaire « SORTIE prématurée/Fin d’étude »)*  🞎AUTRE, précisez la raison :**_____________________________________________________** |

1. **PRISE QUOTIDIENNE DE MEDICAMENTS** (données transcrites à partir de la fiche d’observance complétée par les agents communautaires)

| **TRAITEMENT DE L’ETUDE** | | |
| --- | --- | --- |
| **Date de la visite** [__] [__]/ [__] [__] / [__] [__] [__] [__] | | |
| **Traitement dispensé** ☐OUI ☐NON, si OUI COMPLETER | | |
| **Ciprofloxacine a.m.** | ☐OUI  ☐NON  ☐Inconnu | Dose: [__][__][__][__] mg Voie : ☐IV ☐PO  Si patient suivi à domicile par l’AC :  Dose non prise / vomissements / aggravation de l’état ☐OUI ☐NON  Si OUI Référer le patient au CSB : Patient référé au CSB ☐OUI ☐NON  Si patient suivi au sein de la structure sanitaire :  Remarque sur la prise : |
| **Ciprofloxacine p.m.** | ☐OUI  ☐NON  ☐Inconnu | Dose : [__][__][__][__] mg Voie : ☐IV ☐PO  Si patient suivi à domicile par l’AC :  Dose non prise / vomissements / aggravation de l’état ☐OUI ☐NON  Si OUI Référer le patient au CSB : Patient référé au CSB ☐OUI ☐NON  Si patient suivi au sein de la structure sanitaire :  Remarque sur la prise : |

| **VISITE 8** |
| --- |

1. **VISITE – V8**

| **Date prévisionnelle de la visite** | [__][__]/ [__] [__] / [__] [__] [__] [__] |
| --- | --- |
| **Jour de la visite** | 🞎J1 🞎J2 🞎J3 🞎J4  🞎J5 🞎J6 🞎J7 🞎J8 🞎J9 🞎J10  🞎J11 🞎J21  🞎M3 |
| **Lieu de la visite** | 🞎 Sur site 🞎 A domicile |
| **La visite a –t-elle pu être réalisée ?** | 🞎OUI 🞎NON |
| **Patient vu le jour de la visite, par :** | 🞎 Médecin / Responsable du site  🞎 TEC  🞎 Agent communautaire |
| **Date effective de la visite** | [__][__]/ [__] [__] / [__] [__] [__] [__] |
| **Déclaration évènement indésirable** | 🞎OUI, *si « oui » compléter le formulaire EI*  🞎NON |
| **Si la visite n’a pas pu être réalisée, précisez la raison :** | 🞎 Arrêt prématuré  *(Compléter le formulaire « SORTIE prématurée/Fin d’étude »)*  🞎AUTRE, précisez la raison :**_____________________________________________________** |

1. **PRISE QUOTIDIENNE DE MEDICAMENTS – V8** (données transcrites à partir de la fiche d’observance complétée par les agents communautaires)

| **TRAITEMENT DE L’ETUDE** | | |
| --- | --- | --- |
| **Date de la visite** [__] [__]/ [__] [__] / [__] [__] [__] [__] | | |
| **Traitement dispensé** ☐OUI ☐NON, si OUI COMPLETER | | |
| **Ciprofloxacine a.m.** | ☐OUI  ☐NON  ☐Inconnu | Dose: [__][__][__][__] mg Voie : ☐IV ☐PO  Si patient suivi à domicile par l’AC :  Dose non prise / vomissements / aggravation de l’état ☐OUI ☐NON  Si OUI Référer le patient au CSB : Patient référé au CSB ☐OUI ☐NON  Si patient suivi au sein de la structure sanitaire :  Remarque sur la prise : |
| **Ciprofloxacine p.m.** | ☐OUI  ☐NON  ☐Inconnu | Dose : [__][__][__][__] mg Voie : ☐IV ☐PO  Si patient suivi à domicile par l’AC :  Dose non prise / vomissements / aggravation de l’état ☐OUI ☐NON  Si OUI Référer le patient au CSB : Patient référé au CSB ☐OUI ☐NON  Si patient suivi au sein de la structure sanitaire :  Remarque sur la prise : |

| **VISITE 9** |
| --- |

1. **VISITE – V9**

| **Date prévisionnelle de la visite** | [__][__]/ [__] [__] / [__] [__] [__] [__] |
| --- | --- |
| **Jour de la visite** | 🞎J1 🞎J2 🞎J3 🞎J4  🞎J5 🞎J6 🞎J7 🞎J8 🞎J9 🞎J10  🞎J11 🞎J21  🞎M3 |
| **Lieu de la visite** | 🞎 Sur site 🞎 A domicile |
| **La visite a –t-elle pu être réalisée ?** | 🞎OUI 🞎NON |
| **Patient vu le jour de la visite, par :** | 🞎 Médecin / Responsable du site  🞎 TEC  🞎 Agent communautaire |
| **Date effective de la visite** | [__][__]/ [__] [__] / [__] [__] [__] [__] |
| **Déclaration évènement indésirable** | 🞎OUI, *si « oui » compléter le formulaire EI*  🞎NON |
| **Si la visite n’a pas pu être réalisée, précisez la raison :** | 🞎 Arrêt prématuré  *(Compléter le formulaire « SORTIE prématurée/Fin d’étude »)*  🞎AUTRE, précisez la raison :**_____________________________________________________** |

1. **PRISE QUOTIDIENNE DE MEDICAMENTS – V9** (données transcrites à partir de la fiche d’observance complétée par les agents communautaires)

| **TRAITEMENT DE L’ETUDE** | | |
| --- | --- | --- |
| **Date de la visite** [__] [__]/ [__] [__] / [__] [__] [__] [__] | | |
| **Traitement dispensé** ☐OUI ☐NON, si OUI COMPLETER | | |
| **Ciprofloxacine a.m.** | ☐OUI  ☐NON  ☐Inconnu | Dose: [__][__][__][__] mg Voie : ☐IV ☐PO  Si patient suivi à domicile par l’AC :  Dose non prise / vomissements / aggravation de l’état ☐OUI ☐NON  Si OUI Référer le patient au CSB : Patient référé au CSB ☐OUI ☐NON  Si patient suivi au sein de la structure sanitaire :  Remarque sur la prise : |
| **Ciprofloxacine p.m.** | ☐OUI  ☐NON  ☐Inconnu | Dose : [__][__][__][__] mg Voie : ☐IV ☐PO  Si patient suivi à domicile par l’AC :  Dose non prise / vomissements / aggravation de l’état ☐OUI ☐NON  Si OUI Référer le patient au CSB : Patient référé au CSB ☐OUI ☐NON  Si patient suivi au sein de la structure sanitaire :  Remarque sur la prise : |

| **VISITE 10** |
| --- |

1. **VISITE – V10**

| **Date prévisionnelle de la visite** | [__][__]/ [__] [__] / [__] [__] [__] [__] |
| --- | --- |
| **Jour de la visite** | 🞎J1 🞎J2 🞎J3 🞎J4  🞎J5 🞎J6 🞎J7 🞎J8 🞎J9 🞎J10  🞎J11 🞎J21  🞎M3 |
| **Lieu de la visite** | 🞎 Sur site 🞎 A domicile |
| **La visite a –t-elle pu être réalisée ?** | 🞎OUI 🞎NON |
| **Patient vu le jour de la visite, par :** | 🞎 Médecin / Responsable du site  🞎 TEC  🞎 Agent communautaire |
| **Date effective de la visite** | [__][__]/ [__] [__] / [__] [__] [__] [__] |
| **Déclaration évènement indésirable** | 🞎OUI, *si « oui » compléter le formulaire EI*  🞎NON |
| **Si la visite n’a pas pu être réalisée, précisez la raison :** | 🞎 Arrêt prématuré  *(Compléter le formulaire « SORTIE prématurée/Fin d’étude »)*  🞎AUTRE, précisez la raison :**_____________________________________________________** |

1. **PRISE QUOTIDIENNE DE MEDICAMENTS – V10** (données transcrites à partir de la fiche d’observance complétée par les agents communautaires)

| **TRAITEMENT DE L’ETUDE** | | |
| --- | --- | --- |
| **Date de la visite** [__] [__]/ [__] [__] / [__] [__] [__] [__] | | |
| **Traitement dispensé** ☐OUI ☐NON, si OUI COMPLETER | | |
| **Ciprofloxacine a.m.** | ☐OUI  ☐NON  ☐Inconnu | Dose: [__][__][__][__] mg Voie : ☐IV ☐PO  Si patient suivi à domicile par l’AC :  Dose non prise / vomissements / aggravation de l’état ☐OUI ☐NON  Si OUI Référer le patient au CSB : Patient référé au CSB ☐OUI ☐NON  Si patient suivi au sein de la structure sanitaire :  Remarque sur la prise : |
| **Ciprofloxacine p.m.** | ☐OUI  ☐NON  ☐Inconnu | Dose : [__][__][__][__] mg Voie : ☐IV ☐PO  Si patient suivi à domicile par l’AC :  Dose non prise / vomissements / aggravation de l’état ☐OUI ☐NON  Si OUI Référer le patient au CSB : Patient référé au CSB ☐OUI ☐NON  Si patient suivi au sein de la structure sanitaire :  Remarque sur la prise : |

| **VISITE 11** |
| --- |

1. **VISITE – V11**

| **Date prévisionnelle de la visite** | [__][__]/ [__] [__] / [__] [__] [__] [__] |
| --- | --- |
| **Jour de la visite** | 🞎J1 🞎J2 🞎J3 🞎J4  🞎J5 🞎J6 🞎J7 🞎J8 🞎J9 🞎J10  🞎J11 🞎J21  🞎M3 |
| **Lieu de la visite** | 🞎 Sur site 🞎 A domicile |
| **La visite a –t-elle pu être réalisée ?** | 🞎OUI 🞎NON |
| **Patient vu le jour de la visite, par :** | 🞎 Médecin / Responsable du site  🞎 TEC  🞎 Agent communautaire |
| **Date effective de la visite** | [__][__]/ [__] [__] / [__] [__] [__] [__] |
| **Déclaration évènement indésirable** | 🞎OUI, *si « oui » compléter le formulaire EI*  🞎NON |
| **Si la visite n’a pas pu être réalisée, précisez la raison :** | 🞎 Arrêt prématuré  *(Compléter le formulaire « SORTIE prématurée/Fin d’étude »)*  🞎AUTRE, précisez la raison :**_____________________________________________________** |

1. **SYMPTOMES / SIGNES CLINIQUES – V11**

| **EXAMEN CLINIQUE** | | |
| --- | --- | --- |
| **Date d’évaluation :** [__] [__]/ [__] [__] / [__] [__] [__] [__] **Heure de l'évaluation :** [__] [__]/ [__] [__] | | |
| **Fièvre** | ☐OUI ☐NON ☐Inconnu | |
| **Toux** | ☐OUI ☐NON ☐Inconnu | |
| **Crachats purulents** | ☐OUI ☐NON ☐Inconnu | |
| **Crachats sanglants** | ☐OUI ☐NON ☐Inconnu | |
| **Hémoptysie franche** | ☐OUI ☐NON ☐Inconnu | |
| **Dyspnée** | ☐OUI ☐NON ☐Inconnu | |
| **Douleur thoracique** | ☐OUI ☐NON ☐Inconnu | |
| **Bubon(s)** | ☐OUI Depuis combien de temps en jours [__] [__] Nombre de bubons  [__][__]  ☐NON ☐Inconnu | |
| **Site du Bubon(s)** | ☐Axillaire | ☐OUI ☐NON  ☐ Gauche ☐ Droite  ☐Visible et mesurable ☐Palpable et non mesurable  ☐Ferme ou ☐Souple  Rougeur ☐OUI ☐NON  Œdème des tissus ☐OUI ☐NON  Suppuration ☐OUI ☐NON  Score de douleur ____  Taille  (mm) : Grand Axe [___] [___],[___] [___] Petit Axe [___] [___],[___] [___] |
|  | ☐Cervical | ☐OUI ☐NON  ☐ Gauche ☐ Droite  ☐Visible et mesurable ☐Palpable et non mesurable  ☐Ferme ou ☐Souple  Rougeur ☐OUI ☐NON  Œdème des tissus ☐OUI ☐NON  Suppuration ☐OUI ☐NON  Score de douleur ____  Taille  (mm) : Grand Axe [___] [___],[___] [___] Petit Axe [___] [___],[___] [___] |
|  | ☐Inguinal | ☐OUI ☐NON  ☐ Gauche ☐ Droite  ☐Visible et mesurable ☐Palpable et non mesurable  ☐Ferme ou ☐Souple  Rougeur ☐OUI ☐NON  Œdème des tissus ☐OUI ☐NON  Suppuration ☐OUI ☐NON  Score de douleur ____  Taille  (mm) : Grand Axe [___] [___],[___] [___] Petit Axe [___] [___],[___] [___] |
|  | ☐Autre | ☐OUI ☐NON  ☐ Gauche ☐ Droite  ☐Visible et mesurable ☐Palpable et non mesurable  ☐Ferme ou ☐Souple  Rougeur ☐OUI ☐NON  Œdème des tissus ☐OUI ☐NON  Suppuration ☐OUI ☐NON  Score de douleur ____  Taille  (mm) : Grand Axe [___] [___],[___] [___] Petit Axe [___] [___],[___] [___] |
| **Vomissement** | ☐OUI ☐NON ☐Inconnu | |
| **Diarrhée** | ☐OUI ☐NON ☐Inconnu | |
| **Céphalées** | ☐OUI ☐NON ☐Inconnu | |
| **Crises convulsives** | ☐OUI ☐NON ☐Inconnu | |
| **Niveau de conscience**  **Echelle EODA (AVPU)** | ☐ Eveillé (conscience normale) ☐ Répond aux ordres ☐ Répond aux stimuli douloureux  ☐ Ne répond à aucun stimulus | |
| **PARAMETRES VITAUX** | | |
| **Mesures des signes vitaux** ☐OUI ☐NON **Heure de l’évaluation :** [__] [__]/ [__] [__] | | |
| **Fréquence respiratoire** | [___] [___] cycles / min ☐NF | |
| **Fréquence cardiaque** | [___] [___] [___] pulsations / min ☐NF | |
| **Pression artérielle** | Systolique [___] [___] [___] / mmHg Diastolique [___] [___] [___]/ mmHg ☐NF | |
| **Température axillaire** | [___] [___]. [___] C ☐NF | |
| **Saturation d'oxygène** | [___] [___] % ☐en air ambiant ☐Sur oxygène supplémentaire [___] [___] L / min ☐NF  Débit d’oxygène [___] [___],[___] L / min ☐NF | |

1. **COLLECTE ECHANTILLLONS – V11**

| **Prélèvement sanguin pour la sérologie** | ☐OUI ☐NON, SI « NON », précisez la raison : _____________________________________  Type de prélèvement pour la sérologie  Sang capillaire☐ Sang veineux☐ |
| --- | --- |
|  | Date de prélèvement : [__] [__] / [__] [__] / [__] [__]  **N° Prélèvement SEROLOGIE**  ECR \|__\| \|__\|__\| \|__\|__\| \|__\|__\|__\| \|__\|__\|__\| |

1. **PRISE QUOTIDIENNE DES MEDICAMENTS – V11** (données transcrites à partir de la fiche d’observance complétée par les agents communautaires)

| **TRAITEMENT DE L’ETUDE** | | |
| --- | --- | --- |
| **Date de la visite** [__][__]/ [__] [__] / [__] [__] [__] [__] | | |
| **Traitement dispensé** ☐OUI ☐NON, si OUI COMPLETER | | |
| **Ciprofloxacine a.m.** | ☐OUI  ☐NON  ☐Inconnu | Dose: [__][__][__][__] mg Voie : ☐IV ☐PO  Si la dose manquée, la raison : __________________  Si la dose modifiée, la raison : __________________  Si la dose refusée, la raison : __________________  Si administration de ciprofloxacine IV, préciser la raison  ☐Impossibilité d’administrer la dose PO ☐Autre, précisez :  ______________________________________________________  Autre remarque sur la prise : |
| **Observation sur la prise : vomissements dans les 30 min après la prise** ☐OUI ☐NON, SI OUI COMPLETER | | |
| **Ciprofloxacine ré-administrée (n°1)** ☐OUI ☐NON, Si NON préciser la raison.................................................Si OUI compléter : | | |
|  |  | Dose: [__][__][__][__] mg Voie : ☐IV ☐PO Heure de prise : [__][__]/ [__] [__]  Si la dose manquée, la raison : __________________  Si la dose modifiée, la raison : __________________  Si la dose refusée, la raison : __________________  Si administration de ciprofloxacine IV, préciser la raison  ☐Impossibilité d’administrer la dose PO ☐Autre, précisez :  ______________________________________________________  Autre remarque sur la prise : |
| **Observation sur la prise : vomissements dans les 30 min après la prise** ☐OUI ☐NON, SI OUI COMPLETER | | |
| **Ciprofloxacine ré-administrée (n°2)** ☐OUI ☐NON, Si NON préciser la raison.................................................Si OUI compléter : | | |
|  |  | Dose: [__][__][__][__] mg Voie : ☐IV Heure de prise : [__][__]/ [__] [__]  Si la dose manquée, la raison : __________________  Si la dose modifiée, la raison : __________________  Si la dose refusée, la raison : __________________  Autre remarque sur la prise : |

1. **OBSERVANCE THERAPEUTIQUES A J11**

| **Le patient a-t-il fini les 10 jours de traitement auquel il a été assigné :** ☐OUI ☐NON  Si OUI, date de début : [__] [__]/ [__] [__] / [__] [__] [__] [__]  Date d'arrêt : [__] [__]/ [__] [__] / [__] [__] [__] [__]  Si NON, date de début : [__][__]/ [__] [__] / [__] [__] [__] [__]  Date d'arrêt : [__] [__]/ [__] [__] / [__] [__] [__] [__]  Si NON, quelle était la raison :_____________________  SI NON, Un autre antibiotique a-t-il été utilisé pour traiter la peste : ☐OUI ☐NON  Nom de l'antibiotique :  1.___________________________________________  Date de début : [__] [__]/ [__] [__] / [__] [__] [__] [__]  Date d'arrêt : [__] [__]/ [__] [__] / [__] [__] [__] [__] _]  2. ___________________________________________  Date de début : [__] [__]/ [__] [__] / [__] [__] [__] [__]  Date d'arrêt : [__] [__]/ [__] [__] / [__] [__] [__] [__] |
| --- |
| **Le patient a-t-il continué à prendre des antibiotiques pour le traitement de la peste au-delà de J10** ☐OUI ☐NON  Si OUI, quelle était la raison : ______________________  Nom des antibiotiques :  1. ______________________  2. ______________________  Combien de jours supplémentaires le patient a-t-il reçu :  1. Antibiotique 1 [__] [__]  2. Antibiotique 2 [__] [__] |

| **VISITE 12 (visite de suivi à J21)** |
| --- |

1. **VISITE – V12**

| **Date prévisionnelle de la visite** | [__][__]/ [__] [__] / [__] [__] [__] [__] |
| --- | --- |
| **Jour de la visite** | 🞎J1 🞎J2 🞎J3 🞎J4  🞎J5 🞎J6 🞎J7 🞎J8 🞎J9 🞎J10  🞎J11 🞎J21  🞎M3 |
| **Lieu de la visite** | 🞎 Sur site 🞎 A domicile |
| **La visite a –t-elle pu être réalisée ?** | 🞎OUI 🞎NON |
| **Patient vu le jour de la visite, par :** | 🞎 Médecin / Responsable du site  🞎 TEC  🞎 Agent communautaire |
| **Date effective de la visite** | [__][__]/ [__] [__] / [__] [__] [__] [__] |
| **Déclaration évènement indésirable** | 🞎OUI, *si « oui » compléter le formulaire EI*  🞎NON |
| **Si la visite n’a pas pu être réalisée, précisez la raison :** | 🞎 Arrêt prématuré  *(Compléter le formulaire « SORTIE prématurée/Fin d’étude »)*  🞎AUTRE, précisez la raison :**_____________________________________________________** |

1. **SYMPTOMES / SIGNES CLINIQUES – V12**

| **EXAMEN CLINIQUE** | | |
| --- | --- | --- |
| **Date d’évaluation :** [__][__]/ [__] [__] / [__] [__] [__] [__] **Heure de l'évaluation :** [__] [__]/ [__] [__] | | |
| **Fièvre** | ☐OUI ☐NON ☐Inconnu | |
| **Toux** | ☐OUI ☐NON ☐Inconnu | |
| **Crachats purulents** | ☐OUI ☐NON ☐Inconnu | |
| **Crachats sanglants** | ☐OUI ☐NON ☐Inconnu | |
| **Hémoptysie franche** | ☐OUI ☐NON ☐Inconnu | |
| **Dyspnée** | ☐OUI ☐NON ☐Inconnu | |
| **Douleur thoracique** | ☐OUI ☐NON ☐Inconnu | |
| **Bubon(s)** | ☐OUI Depuis combien de temps en jours [__] [__] Nombre de bubons  [__] [__]  ☐NON ☐Inconnu | |
| **Site du Bubon(s)** | Axillaire | ☐OUI ☐NON  ☐ Gauche ☐ Droite  ☐Visible et mesurable ☐Palpable et non mesurable  ☐Ferme ou ☐Souple  Rougeur ☐OUI ☐NON  Œdème des tissus ☐OUI ☐NON  Suppuration ☐OUI ☐NON  Score de douleur ____  Taille  (mm) : Grand Axe [___] [___],[___] [___] Petit Axe [___] [___],[___] [___] |
|  | Cervical | ☐OUI ☐NON  ☐ Gauche ☐ Droite  ☐Visible et mesurable ☐Palpable et non mesurable  ☐Ferme ou ☐Souple  Rougeur ☐OUI ☐NON  Œdème des tissus ☐OUI ☐NON  Suppuration ☐OUI ☐NON  Score de douleur ____  Taille  (mm) : Grand Axe [___] [___],[___] [___] Petit Axe [___] [___],[___] [___] |
|  | Inguinal | ☐OUI ☐NON  ☐ Gauche ☐ Droite  ☐Visible et mesurable ☐Palpable et non mesurable  ☐Ferme ou ☐Souple  Rougeur ☐OUI ☐NON  Œdème des tissus ☐OUI ☐NON  Suppuration ☐OUI ☐NON  Score de douleur ____  Taille  (mm) : Grand Axe [___] [___],[___] [___] Petit Axe [___] [___],[___] [___] |
|  | Autre | ☐OUI ☐NON  ☐ Gauche ☐ Droite  ☐Visible et mesurable ☐Palpable et non mesurable  ☐Ferme ou ☐Souple  Rougeur ☐OUI ☐NON  Œdème des tissus ☐OUI ☐NON  Suppuration ☐OUI ☐NON  Score de douleur ____  Taille  (mm) : Grand Axe [___] [___],[___] [___] Petit Axe [___] [___],[___] [___] |
| **Vomissement** | ☐OUI ☐NON ☐Inconnu | |
| **Diarrhée** | ☐OUI ☐NON ☐Inconnu | |
| **Céphalées** | ☐OUI ☐NON ☐Inconnu | |
| **Crises convulsives** | ☐OUI ☐NON ☐Inconnu | |
| **Niveau de conscience**  **Echelle EODA (AVPU)** | ☐ Eveillé (conscience normale) ☐ Répond aux ordres ☐ Répond aux stimuli douloureux ☐ Ne répond à aucun stimulus | |
| **PARAMETRES VITAUX** | | |
| **Mesures des signes vitaux** ☐OUI ☐NON **Heure de l’évaluation :** [__] [__]/ [__] [__] | | |
| **Fréquence respiratoire** | [___] [___] cycles / min ☐NF | |
| **Fréquence cardiaque** | [___] [___] [___] pulsations / min ☐NF | |
| **Pression artérielle** | Systolique [___] [___] [___] / mmHg Diastolique [___] [___] [___]/ mmHg ☐NF | |
| **Température axillaire** | [___] [___]. [___] C ☐NF | |
| **Saturation d'oxygène** | [___] [___] % ☐en air ambiant ☐Sur oxygène supplémentaire [___] [___] L / min ☐NF  Débit d’oxygène [___] [___],[___] L / min ☐NF | |

1. **COLLECTE ECHANTILLLONS – V12**

| **Prélèvement sanguin pour la sérologie** | ☐OUI ☐NON  Type de prélèvement pour la sérologie  Sang capillaire☐ Sang veineux☐ |
| --- | --- |
|  | Date de prélèvement : [__] [__] / [__] [__] / [__] [__]  **N° Prélèvement SEROLOGIE**  ECR \|__\| \|__\|__\| \|__\|__\| \|__\|__\|__\| \|__\|__\|__\| |

| **VISITE 13 (visite de suivi à M3)** |
| --- |

1. **VISITE – V13**

| **Date prévisionnelle de la visite** | [__][__]/ [__] [__] / [__] [__] [__] [__] |
| --- | --- |
| **Jour de la visite** | 🞎J1 🞎J2 🞎J3 🞎J4  🞎J5 🞎J6 🞎J7 🞎J8 🞎J9 🞎J10  🞎J11 🞎J21  🞎M3 |
| **Lieu de la visite** | 🞎 Sur site 🞎 A domicile |
| **La visite a –t-elle pu être réalisée ?** | 🞎OUI 🞎NON |
| **Patient vu le jour de la visite, par :** | 🞎 Médecin / Responsable du site  🞎 TEC  🞎 Agent communautaire |
| **Date effective de la visite** | [__][__]/ [__] [__] / [__] [__] [__] [__] |
| **Déclaration évènement indésirable** | 🞎OUI, *si « oui » compléter le formulaire EI*  🞎NON |
| **Si la visite n’a pas pu être réalisée, précisez la raison :** | 🞎 Arrêt prématuré  *(Compléter le formulaire « SORTIE prématurée/Fin d’étude »)*  🞎AUTRE, précisez la raison :**_____________________________________________________** |

1. **COLLECTE ECHANTILLLONS – V13**

| **Prélèvement sanguin pour la sérologie** | ☐OUI ☐ NON  Type de prélèvement pour la sérologie  Sang capillaire☐ Sang veineux☐ |
| --- | --- |
|  | Date de prélèvement : [__] [__] / [__] [__] / [__] [__]  **N° Prélèvement SEROLOGIE**  ECR \|__\| \|__\|__\| \|__\|__\| \|__\|__\|__\| \|__\|__\|__\| |

| **SORTIE HOPITAL** |
| --- |

| **Évolution à la sortie** | ☐Vivant  ☐Transfert vers un autre établissement  ☐Décès  ☐Décharge palliatif  ☐ Retrait prématuré  ☐Autodécharge/sortie contre avis médical  ☐Perdu de vue |
| --- | --- |
| **Date d’évolution** | [__][__]/ [__] [__] / [__] [__] [__] [__] |
| **Date d’évolution à la sortie d’hôpital** | [__][__]/ [__] [__] / [__] [__] [__] [__] |
| **Jour de sortie de la formation sanitaire** | ☐<J4 ☐J4 ☐<J11 ☐J11 |

| **FORMULAIRE SORTIE PREMATUREE / FIN D’ETUDE** |
| --- |

| **Date de sortie prématurée/fin d’étude** | [__][__]/ [__] [__] / [__] [__] [__] [__] |
| --- | --- |
| **Dernière visite réalisée :** | 🞎J1 🞎J2 🞎J3 🞎J4  🞎J5 🞎J6 🞎J7 🞎J8 🞎J9 🞎J10  🞎J11 🞎J21  🞎M3 |
| **Nombre total de visites de suivi effectuées** |  |

| **MOTIF DE LA SORTIE PREMATUREE/FIN D’ETUDE** | |
| --- | --- |
| **Le patient a-t-il suivi la totalité de l'étude ?** | ☐OUI ☐ NON |
| **Si Oui, préciser la raison.**  ***Ne cocher qu’une case (la raison principale)*** | ☐**Inclusion à tort**  Si oui, précisez : _________________________________________________ |
|  | ☐ **Retrait de consentement/ Décision du patient**  Précisez la date: [__] [__]/ [__] [__] / [__] [__] [__] [__]  Préciser  ☐Autorisation d’exploitation des données ☐ OUI ☐NON  ☐Autorisation d’utilisation des échantillons biologiques ☐ OUI ☐NON |
|  | ☐ **Décision médicale**  Si oui, précisez laquelle ________________________ |
|  | ☐ **Décision du Promoteur**  Si oui, précisez laquelle ________________________ |
|  | ☐ **Perdu de vue** (Patient non vu pour les visites de suivi prévues par le protocole)  Si oui, précisez : ☐ Sujet évadé  ☐ Sortie du sujet de l’établissement contre l’avis médical  ☐ Visite J11 non effectuée  ☐ Visite J21 non effectuée  Si oui, précisez la date de dernière nouvelle :  [__][__]/ [__] [__] / [__] [__] [__] [__] |
|  | ☐ **Décès**  Si oui, précisez la date du décès : [__] [__]/ [__] [__] / [__] [__] [__] [__]  Et la cause du décès : _____________________________________________ |
|  | ☐ **Transfert dans un autre centre spécialisé**  Si oui, date du transfert : [__] [__]/ [__] [__] / [__] [__] [__] [__]  Si oui, Diagnostic posé ☐ Non  ☐ Oui, lequel________________________ |
|  | ☐ **Autres**  Précisez : ____________________________________________________ |

| **ÉVÉNEMENTS INDÉSIRABLES** |
| --- |

**En cas d’EI grave : Compléter le formulaire EIG et se référer à la procédure de notification des EIG**

| **EI**  **N ° 1** | **Date à laquelle l’EI a été rapporté** | [__][__]/ [__] [__] / [__] [__] [__] [__] | | | | |
| --- | --- | --- | --- | --- | --- | --- |
|  | **Nom de**  **l'événement** |  | | | | |
|  | **Date d’apparition** | [__][__]/ [__] [__] / [__] [__] [__] [__] ☐Non connue | | | | |
|  | **L’évènement est-il définit comme un EIG** | ☐Oui ☐Non | | | | |
|  | **Grade (sévérité)** | ☐léger ☐Modéré ☐sévère ☐mis en jeu du pronostic vital ☐décès | | | | |
|  | **Lien avec le**  **traitement de l’étude** | Streptomycine | 🞏 Non relié 🞏 Improbable 🞏 Possible 🞏 Probable 🞏 Certain 🞏 Non applicable | | | |
|  |  | Ciprofloxacine | 🞏 Non relié 🞏 Improbable 🞏 Possible 🞏 Probable 🞏 Certain 🞏 Non applicable | | | |
|  | **Evolution** | ☐1, résolu ☐2, résolu avec des séquelles ☐ 3, en cours ☐4, aggravé ☐5, fatal | | | | |
|  | **Date de resolution** | ☐Connue ☐Partiellement connue ☐ Inconnue | | | | |
|  | **Date de resolution** | [__][__]/ [__] [__] / [__] [__] [__] [__] | | | | |
|  | **Date d’aggravation** | [__][__]/ [__] [__] / [__] [__] [__] [__] ☐Non connue | | | | |
|  | **Action sur les**  **médicaments de**  **l’étude** | ☐0, Aucun ☐1, Réduction de dose ☐ 2, Traitement retardé  ☐3, traitement réduit et retardé ☐4, Traitement arrêté définitivement | | | | |
| **EI**  **N ° 2** | **Date à laquelle l’EI a été rapporté** | [__][__]/ [__] [__] / [__] [__] [__] [__] | | | | |
|  | **Nom de**  **l'événement** |  | | | | |
|  | **Date d’apparition** | [__][__]/ [__] [__] / [__] [__] [__] [__] ☐Non connue | | | | |
|  | **L’évènement est-il définit comme un EIG** | ☐Oui ☐Non | | | | |
|  | **Grade (sévérité)** | ☐léger ☐Modéré ☐sévère ☐mis en jeu du pronostic vital ☐décès | | | | |
|  | **Lien avec le**  **traitement de l’étude** | Streptomycine | | | 🞏 Non relié 🞏 Improbable 🞏 Possible 🞏 Probable 🞏 Certain 🞏 Non applicable | |
|  |  | Ciprofloxacine | | | 🞏 Non relié 🞏 Improbable 🞏 Possible 🞏 Probable 🞏 Certain 🞏 Non applicable | |
|  | **Evolution** | ☐1, résolu ☐2, résolu avec des séquelles ☐ 3, en cours ☐4, aggravé ☐5, fatal | | | | |
|  | **Date de resolution** | ☐Connue ☐Partiellement connue ☐ Inconnue | | | | |
|  | **Date de resolution** | [__][__]/ [__] [__] / [__] [__] [__] [__] | | | | |
|  | **Date d’aggravation** | [__][__]/ [__] [__] / [__] [__] [__] [__] ☐Non connue | | | | |
|  | **Action sur les**  **médicaments de**  **l’étude** | ☐0, Aucun ☐1, Réduction de dose ☐ 2, Traitement retardé  ☐3, traitement réduit et retardé ☐4, Traitement arrêté définitivement | | | | |
| **EI**  **N ° 3** | **Date à laquelle l’EI a été rapporté** | [__][__]/ [__] [__] / [__] [__] [__] [__] | | | | |
|  | **Nom de**  **l'événement** |  | | | | |
|  | **Date d’apparition** | [__][__]/ [__] [__] / [__] [__] [__] [__] ☐Non connue | | | | |
|  | **L’évènement est-il définit comme un EIG** | ☐Oui ☐Non | | | | |
|  | **Grade (sévérité)** | ☐léger ☐Modéré ☐sévère ☐mis en jeu du pronostic vital ☐décès | | | | |
|  | **Lien avec le**  **traitement de l’étude** | Streptomycine | 🞏 Non relié 🞏 Improbable 🞏 Possible 🞏 Probable 🞏 Certain 🞏 Non applicable | | | |
|  |  | Ciprofloxacine | 🞏 Non relié 🞏 Improbable 🞏 Possible 🞏 Probable 🞏 Certain 🞏 Non applicable | | | |
|  | **Evolution** | ☐1, résolu ☐2, résolu avec des séquelles ☐ 3, en cours ☐4, aggravé ☐5, fatal | | | | |
|  | **Date de resolution** | ☐Connue ☐Partiellement connue ☐ Inconnue | | | | |
|  | **Date de resolution** | [__][__]/ [__] [__] / [__] [__] [__] [__] | | | | |
|  | **Date d’aggravation** | [__][__]/ [__] [__] / [__] [__] [__] [__] ☐Non connue | | | | |
|  | **Action sur les**  **médicaments de**  **l’étude** | ☐0, Aucun ☐1, Réduction de dose ☐ 2, Traitement retardé  ☐3, traitement réduit et retardé ☐4, Traitement arrêté définitivement | | | | |
| **EI**  **N ° 4** | **Date à laquelle l’EI a été rapporté** | [__][__]/ [__] [__] / [__] [__] [__] [__] | | | | |
|  | **Nom de**  **l'événement** |  | | | | |
|  | **Date d’apparition** | [__][__]/ [__] [__] / [__] [__] [__] [__] ☐Non connue | | | | |
|  | **L’évènement est-il définit comme un EIG** | ☐Oui ☐Non | | | | |
|  | **Grade (sévérité)** | ☐léger ☐Modéré ☐sévère ☐mis en jeu du pronostic vital ☐décès | | | | |
|  | **Lien avec le**  **traitement de l’étude** | Streptomycine | | 🞏 Non relié 🞏 Improbable 🞏 Possible 🞏 Probable 🞏 Certain 🞏 Non applicable | | |
|  |  | Ciprofloxacine | | 🞏 Non relié 🞏 Improbable 🞏 Possible 🞏 Probable 🞏 Certain 🞏 Non applicable | | |
|  | **Evolution** | ☐1, résolu ☐2, résolu avec des séquelles ☐ 3, en cours ☐4, aggravé ☐5, fatal | | | | |
|  | **Date de resolution** | ☐Connue ☐Partiellement connue ☐ Inconnue | | | | |
|  | **Date de resolution** | [__][__]/ [__] [__] / [__] [__] [__] [__] | | | | |
|  | **Date d’aggravation** | [__][__]/ [__] [__] / [__] [__] [__] [__] ☐Non connue | | | | |
|  | **Action sur les**  **médicaments de**  **l’étude** | ☐0, Aucun ☐1, Réduction de dose ☐ 2, Traitement retardé  ☐3, traitement réduit et retardé ☐4, Traitement arrêté définitivement | | | | |
| **EI**  **N ° 5** | **Date à laquelle l’EI a été rapporté** | [__][__]/ [__] [__] / [__] [__] [__] [__] | | | | |
|  | **Nom de**  **l'événement** |  | | | | |
|  | **Date apparition** | [__][__]/ [__] [__] / [__] [__] [__] [__] ☐Non connue | | | | |
|  | **L’évènement est-il définit comme un EIG** | ☐Oui ☐Non | | | | |
|  | **Grade (sévérité)** | ☐léger ☐Modéré ☐sévère ☐mis en jeu du pronostic vital ☐décès | | | | |
|  | **Lien avec le**  **traitement de l’étude** | Streptomycine | 🞏 Non relié 🞏 Improbable 🞏 Possible 🞏 Probable 🞏 Certain 🞏 Non applicable | | | |
|  |  | Ciprofloxacine | 🞏 Non relié 🞏 Improbable 🞏 Possible 🞏 Probable 🞏 Certain 🞏 Non applicable | | | |
|  | **Evolution** | ☐1, résolu ☐2, résolu avec des séquelles ☐ 3, en cours ☐4, aggravé ☐5, fatal | | | | |
|  | **Date de resolution** | ☐Connue ☐Partiellement connue ☐ Inconnue | | | | |
|  | **Date de resolution** | [__][__]/ [__] [__] / [__] [__] [__] [__] | | | | |
|  | **Date aggravation** | [__][__]/ [__] [__] / [__] [__] [__] [__] ☐Non connue | | | | |
|  | **Action sur les**  **médicaments de**  **l’étude** | ☐0, Aucun ☐1, Réduction de dose ☐ 2, Traitement retardé  ☐3, traitement réduit et retardé ☐4, Traitement arrêté définitivement | | | | |
| **EI**  **N ° 6** | **Date à laquelle l’EI a été rapporté** | [__][__]/ [__] [__] / [__] [__] [__] [__] | | | | |
|  | **Nom de**  **l'événement** |  | | | | |
|  | **Date d’apparition** | [__][__]/ [__] [__] / [__] [__] [__] [__] ☐Non connue | | | | |
|  | **L’évènement est-il définit comme un EIG** | ☐Oui ☐Non | | | | |
|  | **Grade (sévérité)** | ☐léger ☐Modéré ☐sévère ☐mis en jeu du pronostic vital ☐décès | | | | |
|  | **Lien avec le**  **traitement de l’étude** | Ciprofloxacine | | | | 🞏 Non relié 🞏 Improbable 🞏 Possible 🞏 Probable 🞏 Certain 🞏 NA |
|  |  | Streptomycine | | | | 🞏 Non relié 🞏 Improbable 🞏 Possible 🞏 Probable 🞏 Certain 🞏 NA |
|  | **Evolution** | ☐1, résolu ☐2, résolu avec des séquelles ☐ 3, en cours ☐4, aggravé ☐5, fatal | | | | |
|  | **Date de resolution** | Connue ☐Partiellement connue ☐ inconnue☐ | | | | |
|  | **Date de resolution** | [__][__]/ [__] [__] / [__] [__] [__] [__] | | | | |
|  | **Date d’aggravation** | [__][__]/ [__] [__] / [__] [__] [__] [__] ☐Non connue | | | | |
|  | **Action sur les**  **médicaments de**  **l’étude** | ☐0, Aucun ☐1, Réduction de dose ☐ 2, Traitement retardé  ☐3, traitement réduit et retardé ☐4, Traitement arrêté définitivement | | | | |
| J'ai examiné les événements indésirables sur cette page et j'en ai évalué la gravité, la causalité, la gravité et les résultats et je confirme qu'au meilleur de ma connaissance, ils reflètent fidèlement l'information obtenue pour ce participant.  **Signature de l’investigateur sur site :**  **Date :** [__][__]/ [__] [__] / [__] [__] [__] [__] | | | | | | |

| **SIGNATURE DE LA DÉCLARATION DE L'INVESTIGATEUR SUR SITE** |
| --- |

| J'ai examiné ce formulaire de cas et je confirme qu'à ma connaissance, il reflète fidèlement l'information sur l'étude obtenue pour ce participant. Toutes les inscriptions ont été faites par moi ou par une personne sous ma supervision qui a signé le registre de délégation et de signature |
| --- |
| **Nom de l’investigateur sur site :** _______________________  **Signature de l’investigateur sur site :** ____________________  **Date :** [__] [__]/ [__] [__] / [__] [__] [__] [__] |

| **RESULTATS LABORATOIRE** |
| --- |

| **Prélèvements** | **Type D’analyses** | **Résultat**  **J1** | | **Résultat**  **J11** | | **Résultat**  **J21** | | **Résultat**  **M3** | |
| --- | --- | --- | --- | --- | --- | --- | --- | --- | --- |
| **Bubon (pus)** | **TDR** | ☐Positif  ☐Négatif  ☐NF | | NA | | NA | | NA | |
|  | **Culture** | ☐Positif  ☐Négatif  ☐NF | | NA | | NA | | NA | |
|  | **PCR** | ☐Positif  ☐Négatif  ☐NF | | NA | | NA | | NA | |
| **Crachat** | **TDR** | ☐Positif  ☐Négatif  ☐NF | | NA | | NA | | NA | |
|  | **Culture** | ☐Positif  ☐Négatif  ☐NF | | NA | | NA | | NA | |
|  | **PCR** | ☐Positif  ☐Négatif  ☐NF | | NA | | NA | | NA | |
| **Sanguin**  **Sérologie (ELISA)** | **IgG** | ☐Positif  ☐Négatif  ☐NF | | ☐Positif  ☐Négatif  ☐NF | | ☐Positif  ☐Négatif  ☐NF | | ☐Positif  ☐Négatif  ☐NF | |
|  | **IgM** | ☐Positif  ☐Négatif  ☐NF | | ☐Positif  ☐Négatif  ☐NF | | ☐Positif  ☐Négatif  ☐NF | | ☐Positif  ☐Négatif  ☐NF | |
| **Sanguin**  **Sérologie (Multiplex)** | **IgG**  **(anti-F1)** | ☐Positif  ☐Négatif  ☐NF | MSI | ☐Positif  ☐Négatif  ☐NF | MSI | ☐Positif  ☐Négatif  ☐NF | MSI | ☐Positif  ☐Négatif  ☐NF | MSI |
|  | **IgM**  **(anti-F1)** | ☐Positif  ☐Négatif  ☐NF | MSI | ☐Positif  ☐Négatif  ☐NF | MSI | ☐Positif  ☐Négatif  ☐NF | MSI | ☐Positif  ☐Négatif  ☐NF | MSI |
